# Microfluidic Chip-LC/MS-based Glycomic Analysis Revealed Distinct *N*-glycan Profile of Rat Serum

**DOI:** 10.1038/srep12844

**Published:** 2015-08-07

**Authors:** Wei-Na Gao, Lee-Fong Yau, Liang Liu, Xing Zeng, Da-Can Chen, Min Jiang, Ju Liu, Jing-Rong Wang, Zhi-Hong Jiang

**Affiliations:** 1 State Key Laboratory of Quality Research in Chinese Medicines, Macau Institute for Applied Research in Medicine and Health, Macau University of Science and Technology, Macau, China; 2 Guangdong Provincial Hospital of Chinese Medicine, Second Clinical College of Guangzhou University of Chinese Medicine, Guangdong Provincial Academy of Chinese Medical Sciences, Guangzhou, China; 3 Division of Rheumatology, Jiujiang First People’s Hospital, Jiujiang, China

## Abstract

The rat is an important alternative for studying human pathology owing to certain similarities to humans. Glycomic studies on rat serum have revealed that variations in the *N*-glycans of glycoproteins correlated with disease progression, which is consistent with the findings in human serum. Therefore, we comprehensively characterized the rat serum *N*-glycome using microfluidic chip-LC-ESI-QTOF MS and MS/MS techniques. In total, 282 *N*-glycans, including isomers, were identified. This study is the first to present comprehensive profiling of *N*-glycans containing *O-*acetylated sialic acid, among which 27 *N*-glycans are novel. In addition, the co-existence of *N*-acetylneuraminic acid (NeuAc) and *N-*glycolylneuraminic acid (NeuGc) in a single *N-*glycan (‘mixed’ *N-*glycan) was detected and represents a new type of *N-*glycan in rat serum. The existence of *O-*acetylated sialic acid is the characteristic feature of rat serum that distinguishes it from mouse and human sera. Comparisons between the rat, mouse, and human serum glycomes revealed that the rat glycome is more similar to that of human sera than to that of mouse sera. Our findings highlight the similarities between the glycomic profile of rat and human sera and provided important selection criteria for choosing an appropriate animal model for pathological and pharmacological studies.

Owing to the inherent similarity to humans, rats have been used as subjects for scientific research in many experimental studies that have contributed significantly to our understanding of a variety of scientific areas including genetics, human diseases, and pharmacological studies. Among rodents, rats are physiologically most similar to humans. For instance, the number of detoxifying P450 gene in rats is similar to that in humans than to that in mice, which explains superiority of rats over mice in pharmacologic investigations^1^. Besides, ribosomal protein genes, which play important role to build up ribosomes, have high sequence similarity between rat and human^2^. A rat model is therefore superior to a mouse model for assaying the pharmacodynamics and toxicities of drugs^1^ and for studying various human diseases, such as cardiovascular disease, arthritis, diabetes mellitus, and autoimmune disorders^3^, and numerous rat models have been established for such types of *in vivo* research.

In the post-genomic era, glycosylation has been brought to public attention as a frontier^4^. The *N-*glycans of glycoproteins are directly involved in almost every biological process and play a crucial role in human diseases due to their unusual biological selectivities^5^. Consequently, glycomics, which is one of the latest members in the “-omics” family of the life sciences, has started to gain its respectable role based on its substantially impact on research towards diagnosing, preventing, and treating diseases^6^,^7^. For example, differences in the glycosylation of serum proteins between cancer patients and healthy people have been distinguished for potential biomarker identification^8^. These non-invasive *N-*glycan biomarkers are promising for disease prognosis. As it is very important to develop reliable strategies to monitor progression of disease in the animal model, the glycomic investigation by using animal model is a necessity. Therefore, glycomics-based studies using animal models have been increasingly reported. For example, glycomic studies of liver cancer in rats revealed that variations in fucosylation of *N-*glycans in serum glycoproteins was closely related to the progression of hepatocellular carcinomas. This was consistent with observations for human liver cancer^9^,^10^,^11^. However, in comparison to the vast array of research into the human glycome, limited studies have been performed on the rat glycome. Less than 50 *N-*glycan compositions of rat serum have been reported to date^9^,^10^. Therefore, a comprehensive glycomic study in rats is much needed. The findings from which may aid our understanding of the physiology and pathology associated with glycosylation for various diseases.

Although direct measurements of free *N-*glycans may seem to be limited in that the information associated with the structure of the glycoprotein is lost owing to the deglycosylation step, such measurements are still significative because: (1) *N-*glycans are often the crucial functional elements in cellular and biomolecular interactions; (2) glycomic techniques are methodologically easier than the glycoproteomic approaches 5.To carry out a comprehensive glycomic study, the primary analytical challenge is the detection and identification of rat-specific *N-*glycans, especially those in low abundance and unknown *N-*glycans. The analytical method must therefore display high detection sensitivity. Moreover, because of the inherent complexity of *N-*glycans, numerous *N-*glycans with similar structures might be co-eluted, leading to significant ionization suppression and a compromised MS/MS spectrum. Therefore, highly efficient separation of *N-*glycans is also imperative. In this study, we developed a microfluidic porous graphitized carbon (PGC) chip-LC/MS-based approach for the profiling of rat serum *N-*glycans. As a type of nano-LC, this microfluidic-chip has many advantages over routine HPLC and UHPLC^12^,^13^. First, the microfluidic chip-LC shows favorable mass spectrometric sensitivity because of reduced flow rates, minimized dead volume, and lower peak dispersion^14^. Second, the PGC-based stationary phase enables isomer-specific separation of *N-*glycans, and thus facilitates maximum resolution of the overlapping ions^15^. Therefore, employment of this microfluidic PGC chip-LC/MS technique in glycomic studies would provide high sensitivity and high resolution. Notably, in this study, higher pH and high ionic strength mobile phase instead of routine mobile phase was applied to PGC-Chip for the first time, chromatographic and MS conditions were then carefully optimized, leading to remarkable improvement in the detection of sialylated *N-*glycans, especially multi-sialylated *N-*glycans. This report describes the comprehensive glycome profiling of rat serum using this newly developed technique and then reports glycomic comparisons of rat, mouse, and human sera.

## Results

### Preparation of *N-*glycan sample for glycomic profiling

The initial step for *N-*glycan analysis is the release of *N-*glycans from glycoproteins. Enzymatic hydrolysis using peptide *N-*glycosidase F (PNGase F) is the most frequently used method for this step. A variety of protocols with varied digestion buffers, denaturation conditions, and digestion times have been employed in previous studies^16^,^17^. To select the conditions that allow for highly efficient release of *N-*glycans without affecting their stability, the three most frequently used protocols were selected for comparison, these included: (1) digestion with thermal denaturation in SDS-containing buffer, with the addition of DTT^18^; (2) digestion in ammonium bicarbonate buffer^16^,^17^; (3) digestion with thermal denaturation in ammonium bicarbonate buffer^19^. The results indicated that the intensities of acidic *N-*glycans varied greatly under different digestion conditions, while those of neutral *N-*glycans obtained by different methods were relatively stable (Supplementary Figure S1). Detailed comparison of individual *N-*glycans suggested that acidic *N-*glycans with one sialic acid were more sensitive to the digestion conditions than those with two or more sialic acids. Hence, serum samples were enzymatically digested using Method (1) in the following experiments.

The next step in the process is purification of the released *N-*glycans from the digestion mixture. In the digestion mixture, de-glycosylated proteins and various salts need to be removed prior to LC-MS analysis so as to avoid contamination of the chip and ion suppression of the signal^20^. These procedures are especially crucial for the nano-LC system, because the proteins and salts may precipitate to block the micro-column in the chip and the spray needle^21^. Purification of *N-*glycans was performed using four different strategies: a PGC cartridge^19^,^22^,^23^, a reverse-phase cartridge^24^,^25^, ultrafiltration^26^, and precipitation^27^. The results were evaluated based on the recovery rate of both neutral and acidic *N-*glycans. The highest recovery rate was achieved using the PGC method, and neutral and acidic *N-*glycans were both sensitive to the enrichment methods (Supplementary Figure S2). Moreover, the PGC method is more efficient than the other methods because salt, *N-*glycans, and de-glycosylated proteins can be sequentially eluted from the PGC cartridge^23^. Given the labile property of sialylated *N-*glycan during the sample preparation, chromatographic separation and ionization in MS^28^,^29^, the stability of di-sialylated *N-*glycan was determined in our experiment with varied concentration. The results suggested that native sialylated *N-*glycans are quite stable when using the method described. Thus the determined *N-*glycans represent a “genuine” and “primary” profile of rat serum (Data not show).

### Separation of *N-*glycans on the microfluidic PGC chip

When using routine mobile phases, *i.e.*, 0.1% formic acid in water and acetonitrile (pH 3), highly sialylated *N-*glycans (containing three or four sialic acids) were out of the scope of analysis due to their poor peak shape^30^, leading to the missed detection of important *N-*glycans. To improve the detection of these highly sialylated *N-*glycans, several mobile phases with varied pH values and ionic strengths were compared. As a result, 0.5% formic acid of pH 3.0 (adjusted by adding ammonium hydroxide) in water and acetonitrile was shown to be the optimal mobile phase for the separation of acidic *N-*glycans. A significantly improved peak shape for all acidic *N-*glycans, especially the highly sialylated *N-*glycans, was obtained using this mobile phase. In addition, the pH of this mobile phase allowed separation of acidic *N-*glycans from neutral *N-*glycans, thus avoiding ion suppression, which in turn enhanced the sensitivity of detection of acidic *N-*glycans. Furthermore, the improved peak shape further facilitated resolution of several groups of sialylated *N-*glycan isomers on the PGC nano-column. For example, the two peaks corresponding to isomers of Hex_5_HexNAc_4_NeuAc_2_ were observed under the developed chromatographic conditions (Supplementary Figure S3). With the good separation of *N-*glycans, several consistent trends in the retention times of *N-*glycans on the PGC stationary phase were observed and are summarized in Supplementary Figures S4 and S5. These retention behaviors provided additional information for the assignment of *N-*glycans. In addition, the repeatability of *N-*glycan profiles as analyzed by our platform was high (Supplementary Figure S6). This study indicated that chip-based-LC/MS technology is a stable technology suitable for the comparative study of the *N-*glycan profiles of rat, mouse, and human sera.

### Methods for the comprehensive characterization of rat serum *N-*glycans

**Figure 1:**
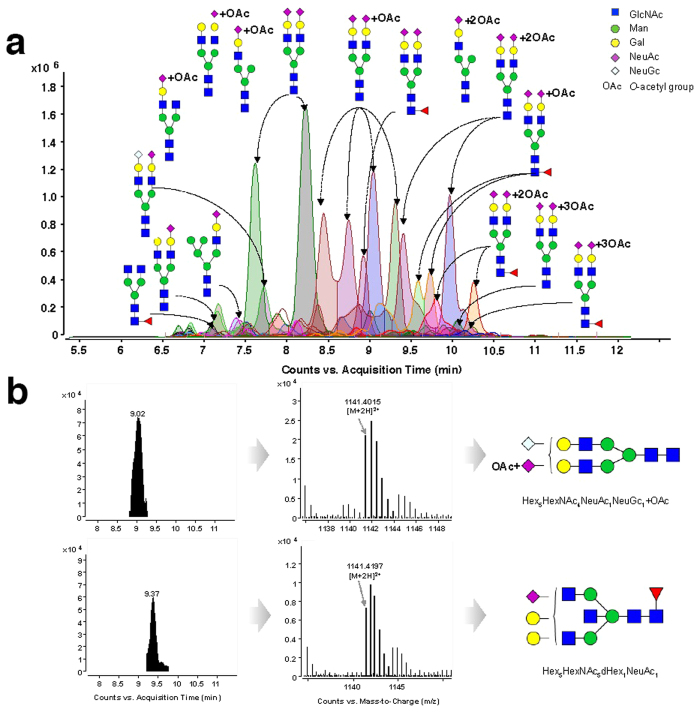
Overlaid extracted compound chromatograms (ECC) of rat serum *N-*glycans identified by PGC nano-LC/MS (a) and differentiation of isobaric *N-*glycans based on the accurate mass value (b).

With the improved separation and detection, the raw data were analyzed on the basis of retention time, accurate mass, isotope abundance, and MS/MS fragmentation. In total, 282 *N-*glycans including 192 acidic *N-*glycans and 90 neutral *N-*glycans were identified, among which 172 *N-*glycans were reported from rat serum for the first time, and 27 were novel *N-*glycans (Fig. 1a and Supplementary Table S1). The analytical procedure is described step-by-step below.

#### Characterization of N-glycans based on accurate mass

For the characterization of *N-*glycans, a MassHunter Personal Compound Database and Library (PCDL) was created. This theoretical *N-*glycan database was established based on the knowledge of mammalian serum *N-*glycan biosynthesis and the possible modifications^9^,^10^,^19^,^31^. As a result, a theoretical library of rat serum *N-*glycans consisting of more than 4000 *N-*glycan compositions was created for identification purposes.

For the determination of *N-*glycan composition, accurate mass and isotopic clusters for each compound were matched with the theoretical mass values of *N-*glycans in the PCDL. The ultra-high accurate mass value obtained by Q-TOF MS facilitated assignment of *N-*glycans at the MS level. In particular, isobaric *N-*glycans can be well differentiated based on the high-resolution MS data. For example, the *N-*glycan at *m/z* 1141.4015 was identified as disialylated *N-*glycan Hex_5_HexNAc_4_NeuAc_1_NeuGc_1_ + OAc based on its measured mass value (C_86_H_140_N_6_O_64_, theoretical mass 2280.7885), while the *N-*glycan at *m/z* 1141.4197 was characterized as Hex_5_HexNAc_5_dHex_1_NeuAc_1_ on the basis of its measured mass value (C_87_H_144_N_6_O_63_, theoretical mass 2280.8247). Both *N-*glycans could be unambiguously distinguished according to their accurate mass values (Fig. 1b).

#### MS/MS screening for NeuAc- and NeuGc-containing N-glycans

After each *N-*glycan was assigned based on its accurate mass, the monosaccharide compositions were confirmed using targeted MS/MS experiments. *N-*glycolylneuraminic acid (NeuAc) and *N-*glycolylneuraminic acid (NeuGc) represent two of the most common sialic acids in nature. *N-*glycans containing NeuAc and NeuGc were widespread on all mammalian cell surfaces in a species-specific manner^19^,^32^,^33^,^34^,^35^, and were associated with pathological conditions^36^,^37^,^38^. This indicates the importance of identification of NeuAc and NeuGc. For organisms expressing both NeuAc and NeuGc, such as rat, it is necessary to differentiate these two species on the basis of chemical evidence.

**Figure 2:**
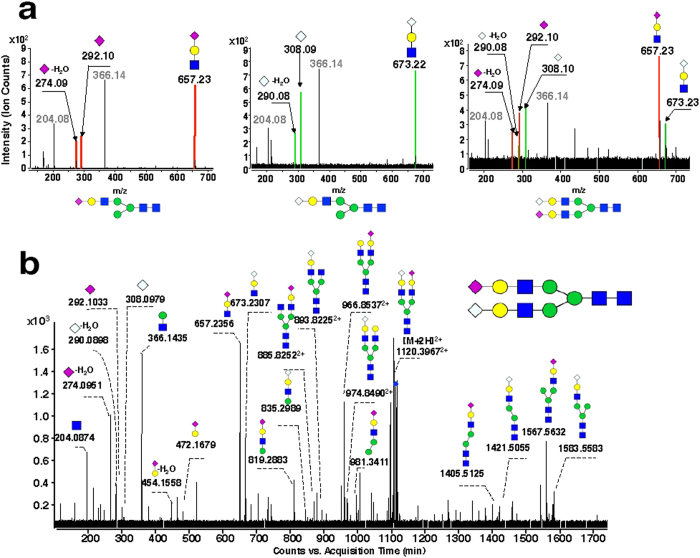
Targeted MS/MS of sialylated *N-*glycans in rat serum. (**a**) Characteristic fragment ions derived from *N-*glycans containing NeuAc (Left), NeuGc (Middle), and both NeuAc and NeuGc (Right). (**b**) Full MS/MS spectra of rat serum *N-*glycan Hex_5_HexNAc_4_NeuAc_1_NeuGc_1_ (doubly protonated *m*/*z* 1120.40).

The only difference between the two sialic acids, NeuAc and NeuGc, is an additional oxygen atom in the *N-*glycolyl group of NeuGc (acetyl amino group)^39^. Such subtle differences in the molecular composition led to the formation of a series of isomers of these two species. For example, the molecular formula of Hex_4_HexNAc_3_NeuAc_1_ (C_59_H_98_N_4_O_44_) is exactly the same as that of Hex_3_HexNAc_3_dHex_1_NeuGc_1_ (C_59_H_98_N_4_O_44_). For differentiating these types of isomers, targeted MS/MS experiments were carried out. Fragment ions derived from respective sialic acids, *i.e*., product ions at *m/z* 274.09 (NeuAc-H_2_O), *m/z* 292.10 (NeuAc), and *m/z* 657.23 (Hex_1_HexNAc_1_NeuAc_1_) generated from NeuAc-containing *N-*glycan, as well as fragment ions at *m/z* 290.08 (NeuGc-H_2_O), *m/z* 308.09 (NeuGc), and *m/z* 673.22 (Hex_1_HexNAc_1_NeuGc_1_) derived from NeuGc-containing *N-*glycan, were employed as diagnostic ions for differentiation of these two species (Fig. 2a). This approach facilitated the identification of a large number of sialylated *N-*glycans, among which 129 were NeuAc-containing *N-*glycans, 59 were NeuGc-containing *N-*glycans, and four were ‘mixed’ *N-*glycans containing both NeuAc and NeuGc (Fig. 2b). Among the four mixed *N-*glycans, *i.e*., Hex_5_HexNAc_4_NeuAc_1_NeuGc_1_, Hex_5_HexNAc_4_NeuAc_1_NeuGc_1_ + OAc, Hex_5_HexNAc_4_dHex_1_NeuAc_1_NeuGc_1_, and Hex_5_HexNAc_4_dHex_1_NeuAc_1_NeuGc_1_ + OAc, only Hex_5_HexNAc_4_NeuAc_1_NeuGc_1_ was found on bovine plasminogen and porcine vitronectin^40^,^41^ and mice serum^42^. This is the first time that this type of sialylated *N-*glycan has been identified in rats.

#### Screening for modified N-glycans

Generally, additional modifications of *N-*glycans occur on the hydroxyl group of the monosaccharide, these may include *O*-acetylation, methylation, lactylation, sulfation, or phosphorylation^43^,^44^,^45^. These modifications result from the actions of different transferases and are monosaccharide specific. For example, mannose is often modified by phosphorylation^44^,^46^, while galactose and *N-*acetylhexosamine are generally modified by sulfation^45^,^47^. Notably, modification of sialic acid is not only the most abundant but also the most structurally diverse because all of the aforementioned modifications can occur on sialic acid^43^.

**Figure 3:**
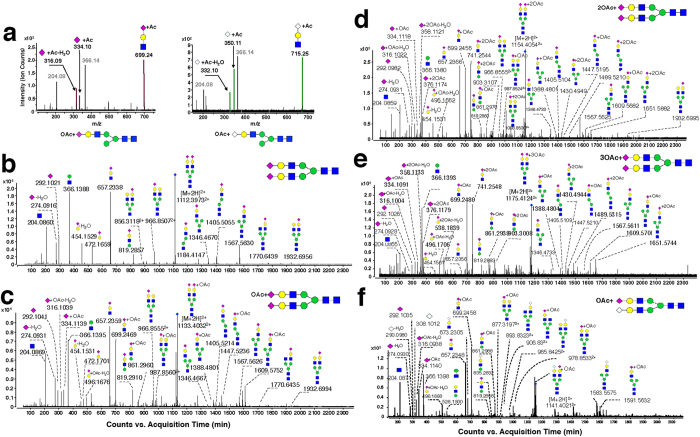
Targeted MS/MS of *O-*acetylated *N-*glycans in rat serum. (**a**) Diagnostic fragment ions generated from *N-*glycans with *O-*acetylated NeuAc (Left) and *O-*acetylated NeuGc (Right). (**b**–**e**) Full MS/MS spectra of a series of *N-*glycans with varied numbers (0–3) of acetylation sites on NeuAc and (**f**) *N-*glycan Hex_5_HexNAc_4_NeuAc_1_ NeuGc_1_ + OAc.

To survey the modifications of rat serum *N-*glycans, the aforementioned five types of modifications were all included in the PCDL for characterization. On the basis of accurate mass, isotopic abundance, and targeted MS/MS, *O-*acetylation was found to be the dominant modification of rat serum *N-*glycans. *O-*acetylation occurred on the terminal sialic acid, which has only be reported in the serum of Atlantic salmon^48^ and several glycoproteins of rats^31^,^49^. Taking di-sialylated *N-*glycan Hex_5_HexNAc_4_NeuAc_2_ and its mono-*O-*acetylation as an example, the identification procedures were performed as follows. First, *N-*glycans were identified based on PCDL. Because the accurate mass and isotopic abundance of compounds in the MS scan were highly similar to the theoretical data on PCDL, they were preliminarily identified as Hex_5_HexNAc_4_NeuAc_2_ and Hex_5_HexNAc_4_NeuAc_2_ + OAc. Then, *O-*acetylation was verified by analysis of an associated fragment produced by targeted MS/MS. Regular variations were found by comparing the MS/MS spectra of the two *N-*glycans (Fig. 3a). A series of fragments, such as *m/z* 274.09, 292.10, and 657.23, shown in the Hex_5_HexNAc_4_NeuAc_2_ MS/MS spectrum (Fig. 3b) indicated the existence of HexNAc + Hex + NeuAc, that is sequentially attached to the core structure of the *N-*glycan. For Hex_5_HexNAc_4_NeuAc_2_ + OAc (Fig. 3c), the series of fragments were 42 Da more than the corresponding fragments in Hex_5_HexNAc_4_NeuAc_2_, *i.e*., *m/z* 316.09 (274.09 + 42), 334.10 (292.10 + 42), and 699.24 (657.23 + 42). These characteristic ions facilitated identification of *O-*acetylated sialic acid in *N-*glycans. Using this approach, *N-*glycans Hex_5_HexNAc_4_NeuAc_2_ with between one and three acetyl groups were identified (Fig. 3d,e).

It is worth mentioning that uncommon disialylated *N-*glycans with three *O-*acetyl groups, Hex_5_HexNAc_4_NeuAc_2_ + 3OAc and Hex_5_HexNAc_4_dHex_1_NeuAc_2_ + 3OAc, were identified in rat serum using this approach. In these *N-*glycans, one *O-*acetyl group was located at one terminal NeuAc, while the other two *O-*acetyl groups were located at another terminal NeuAc. This approach also led to the identification of a novel *O-*acetylated ‘mixed’ *N-*glycan possessing both NeuAc and NeuGc in the molecule, *i.e.*, Hex_5_HexNAc_4_NeuAc_1_NeuGc_1_ + OAc (*m*/*z* 1141.40) (Fig. 3f). Acetylation-associated fragments are summarized in Table 1 and the characterized *O-*acetylated *N-*glycans of rat serum are listed in Table 2. It was noted that, although both NeuAc and NeuGc exist in rat serum *N-*glycans, *O-*acetylation exclusively occurs on NeuAc.

### Comparison of the *N-*glycomic profiles of rat, mouse, and human sera

**Figure 4:**
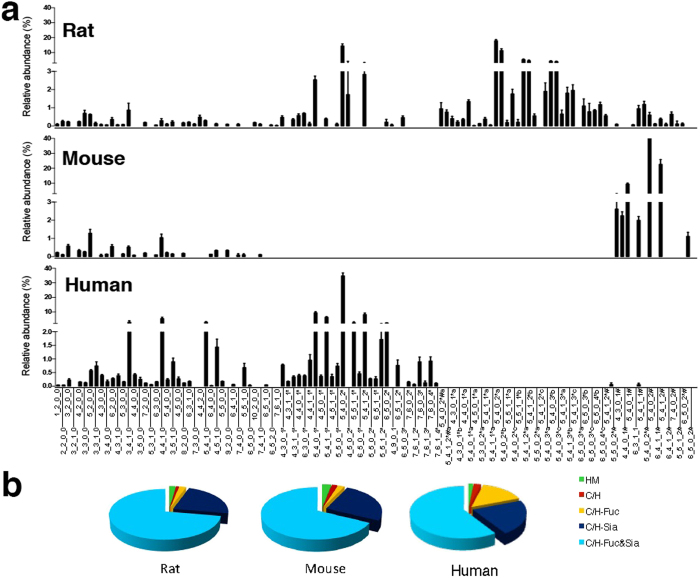
The overall relative abundances of 97 distinct *N-*glycan compositions in rat, mouse, and human sera (a) and relative abundances of each *N-*glycan class. (**b**) High mannose (HM), undecorated complex/hybrid (C/H), fucosylated complex/hybrid (C/H-Fuc), sialylated complex/hybrid (C/H-Sia), and fucosylated/sialylated complex/hybrid (C/H-Fuc&Sia). For the full name of each *N-*glycan refer to Supplementary Table S1.

**Figure 5:**
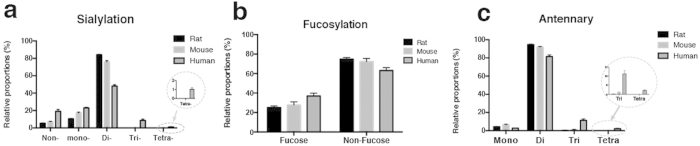
Species-specific variations of *N-*glycans with different numbers of sialic acids, (a) with or without fucose, (b) and with different numbers of antennary, (c) in rat, mouse, and human sera.

According to different biosynthetic procedures, serum *N-*glycans were divided into five classes: high mannose (high Man) *N-*glycans; undecorated complex/hybrid (C/H) *N-*glycans; fucosylated complex/hybrid (C/H-F) *N-*glycans; sialylated complex/hybrid (C/H-S) *N-*glycans; and fucosylated-sialylated complex/hybrid (C/H-FS) *N-*glycans. Using the established glycomic approach, *N-*glycans of mouse and human sera were also comprehensively profiled and compared with *N-*glycans of rat serum (Fig. 4a). Relative abundances were assigned to each *N-*glycan class on the basis of the total ion counts for the individual *N-*glycan signals (Fig. 4b). The results showed that sialylated *N-*glycans were much more abundant than neutral *N-*glycans in all three species, accounting for about 70–80% of the total *N-*glycans in the serum of each species. Di-sialylated *N-*glycans were always the most dominant species, accounting for more than 50% of the total *N-*glycans, followed by mono-, tri-, and tetra-sialylated *N-*glycans (Fig. 5a). In addition, fucosylated *N-*glycans (25–40%) were significantly less abundant than their counterparts (60–75%) in the sera of all three species (Fig. 5b), and more than 80% of the *N-*glycans in rat, mouse, and human sera possessed di-antennary structures, whereas mono- and tri-antennary structures were less abundant (Fig. 5c).

**Figure 6:**
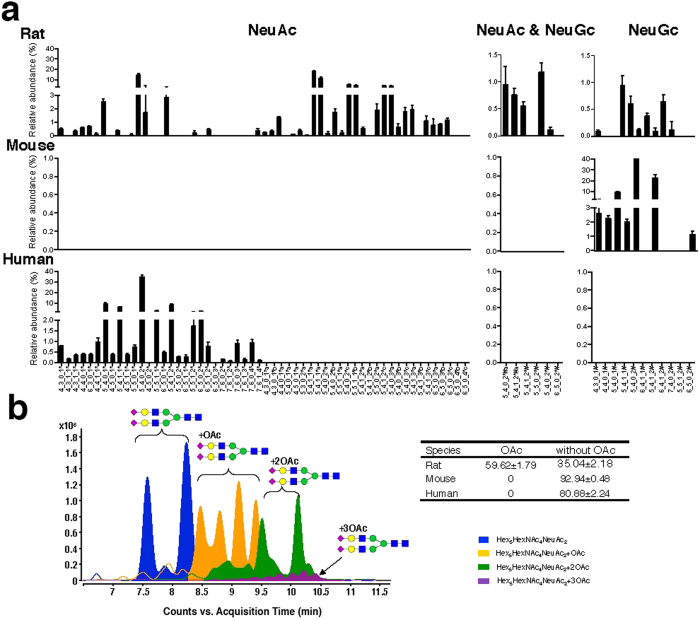
Variant relative abundance of *N-*glycans with NeuAc or NeuGc, or with both NeuAc and NeuGc in rat, mouse, and human sera (a) and the retention behavior of *O-*acetylated *N-*glycans on the PGC chip (**b**) Insert table is the relative abundance of *O-*acetylated *N-*glycans in three species sera.

Despite similarities in the total levels of *N-*glycans in sera among the three species, the differences in the composition of serum *N-*glycans were remarkable among rats, mice, and humans. First, the most notable difference in *N-*glycans among rat, mouse, and human sera was the type of sialic acid present (Fig. 6a). In humans, sialic acids in the serum *N-*glycans were exclusively in the form of NeuAc, whereas NeuGc was the sole sialic acid moiety in mouse serum. However, both NeuAc and NeuGc were identified in the *N-*glycans of rat serum. It is noted that the content of NeuAc in rat serum is almost 20 times that of NeuGc, which is quit similar to human glycome in which NeuAc is the solely acidic species. Second, a large number of sialylated *N-*glycans with *O-*acetylation were detected in rat serum, which, by contrast, were of low abundance in human serum and extremely low abundance in mouse serum (Fig. 6b). The distinct *O-*acetylation of rat glycome was structurally a kind of modification, and this modification exclusively occurred on NeuAc. Our results indicated a species-specific composition of sialic acid in the *N-*glycans of human, mouse and rat sera. However, the glycomic profile of rat serum was more similar to that of human serum based on both the high abundance of NeuAc and similar glycomic distributions (Fig. 4a).

## Discussion

A comprehensive glycomic study of rat serum is not available in the literature. To date, less than 50 *N-*glycan compositions have been reported, significantly less than those identified in human sera^50^,^51^ and mouse sera^19^ (about 100 compositions for each species). Using optimized chromatographic conditions, overlapping *N-*glycans were resolved as far as possible on the PGC-chip, and ionization suppression was significantly reduced, thereby allowing for the detection of low abundance *N-*glycans. Another advantage of the optimized chromatographic conditions was the improved separation of highly sialylated *N-*glycans. Fourteen tri- and two tetra-sialylated *N-*glycans that could not be detected using routine methods were fully characterized in this study. Collectively, our microfluidic chip-LC-MS-based approach enabled improved detection of both neutral and acidic *N-*glycans with highly enhanced sensitivity of the low abundance *N-*glycans. This approach resulted in the detection of 282 *N-*glycans, including 27 novel *N-*glycans in rat serum.

In addition to the overall enhanced sensitivity, employment of microfluidic chips also allowed the separation of isomeric/isobaric species. Efficient separation of the isomeric/isobaric *N-*glycans is crucial for the acquisition of the target MS/MS spectrum, which is in turn essential for the differentiation of these isomeric/isobaric species. In the current study, isomeric species of NeuAc and NeuGc were successfully discriminated in rat serum. This was a significant finding as it is the first report that both types of sialic acid exist in single *N-*glycans in this animal model.

Overall enhanced sensitivity, separation-facilitated reliable MS/MS experiments, together with annotation on the basis of well-established PCDL, further led to the discovery of 102 *N-*glycans containing *O-*acetylated sialic acid in rat serum. Of these, 27 *O-*acetylated *N-*glycans from 12 compositions were reported for the first time in rat serum. The extensive *O-*acetylation of *N-*glycans is clearly a distinguishing feature of rat serum, as the *O-*acetylation level of sialylated *N-*glycans in mouse and human sera is extremely low. *O-*acetylation, the most common modification of sialic acid^52^, has been suggested to be involved in a variety of biological processes in mammals, such as lectin recognition, virus binding, tumor antigenicity, tissue morphogenesis, and cell-cell interactions^43^,^53^. The implications of the extensive *O-*acetylation of *N-*glycans detected in rat serum need to be explored in future studies.

The glycomic analysis carried out in this study provided a comprehensive *N-*glycan profile of rat serum. The results revealed a high abundance of acidic *N-*glycans containing NeuAc and/or NeuGc. The degree of sialylation varied from one to four, with di-sialylated *N-*glycans as the most dominant species. Significantly, structural analysis of the rat serum *N-*glycans revealed extensive *O-*acetylation of NeuAc, which was more diverse than any previous report^30^,^48^. The degree of *O-*acetylation at distinct sites varied from one to three, with mono-acetylation being the most dominant species. To the best of our knowledge, this is the first report showing the extensive *O-*acetylation of the *N-*glycome of rat serum.

Animal models have been used increasingly for investigations in the field of glycobiology. Such investigations are based on the assumption that high similarity exists between the glycomes of mice or rats and humans, which has not yet been extensively studied. The aim of the current study was therefore to carry out a comprehensive comparison of the glycome of rat, mouse, and human sera. Similarities in the serum glycome of these three species were revealed based on the patterns of sialylation, fucosylation, and the degree of branching of *N-*glycans. This is ascribable to the fact that the biosyntheses of *N-*glycans are primarily regulated by hundreds of transferases encoded by the same genes in all three mammals^54^,^55^.

However, there were some notable differences in the types of sialic acid modifications on the *N-*glycans of these three species, *i.e.,* NeuAc, NeuGc, and *O-*acetylated NeuAc. These subtle differences between species may be explained by evolution and epigenetic regulation. First, because the CMAH gene mutation in the human genome occurred by a lethal microbial pathogen that required cell-surface NeuGc for effective infection, humans are now deficient in NeuGc biosynthesis^56^, whereas such a mutation is rare in other vertebrates^57^. Thus, species-specific sialic acids existed: NeuAc for human sera, NeuGc for mouse sera, and both NeuAc and NeuGc for rat sera. Second, glycosylation is not a simple addition to a protein, but an intricately and carefully regulated physiological process, which is affected by epigenetic regulation, which includes competing enzyme activities and the availability of activated monosaccharide donors^58^,^59^,^60^. Epigenetic regulation can modify biological structures and add/remove covalent groups, such as *O-*acetyl and methyl groups, which play important roles in the adaptation to specific environmental conditions^61^,^62^. Among these modifications, *O-*acetylation plays a crucial role in the regulation of ligand function of terminal sialic acids on *N-*glycans^63^. It is well established that the attachment of influenza A and B viruses can be inhibited by *O-*acetylation of sialylated ligands owing to reducing viral sialidase activity^48^,^63^. Thus, it could be speculated that the unique and extensive *O-*acetylation of *N-*glycans in rat serum may be partially associated with the strong immune resistance of rats against various ailments^64^. Despite the distinct features of rat serum glycome, it was more similar to human serum glycome than the mouse serum glycome. In conclusion, our results provide important glycomic data that will aid selection of the most appropriate animal model for pathological and pharmacological studies.

## Methods

This study was approved by the Ethics Committee of the Guangdong Provincial Chinese Medicine Hospital. The methods were carried out in accordance with the approved guidelines.

### *N-*glycan nomenclature and abbreviations

Nomenclature and cartoons of *N-*glycan species are presented in accordance with the Consortium for Functional Glycomics.

### Materials and chemicals

Peptide *N-*glycosidase (PNGase F) was purchased from New England Biolabs (Ipswich, MA, USA). Dithiothreitol (DTT) was purchased from GE Healthcare (Uppsala, Sweden), HyperSep Hypercarb PGC was from Thermo Scientific (Runcorn WA7 1TA, UK). Sep-Pak Vac (1cc) was obtained from Waters (Milford, MA, USA). Centrifuge filters for ultrafiltration were a product of Millipore (Carrigtowohill, Ireland). Acetonitrile (LC/MS grade) and water were from Avantor (Center Valley, PA, USA), formic acid and ammonium bicarbonate (LC/MS grade) were products of Sigma-Aldrich (St. Louis, MO, USA). Reagent grade ammonia and acetone were obtained from Scharlab S. L (Sentmenat, Spain).

### Collection of serum samples

Sera of male Sprague-Dawley rats weighing 200–250 g and ICR male mice weighing 20–22 g were collected via orbital eye bleeding. Sera from volunteers were obtained from the Guangdong Provincial Chinese Medicine Hospital.

### Sample preparation

*N-*glycan release and purification were performed according to the PNGase F protocol provided by New England Biolabs (Ipswich, MA, USA). Briefly, 5 μL of serum were thermally denatured in denaturing buffer containing 0.5% SDS and 40 mM DTT, 10 min prior to digestion by PNGase F (New England Biolabs). Digestion was performed in 50 mM sodium phosphate and 1% NP-40 overnight at 37 °C. Released *N-*glycans were purified by Hypercarb solid-phase extraction (SPE) cartridges, washed with water, then eluted with 40% acetonitrile and 0.05% trifluoroacetic acid (v/v) in water^19^,^22^,^23^. The samples were dried *in vacuo* before MS analysis.

### Chromatographic separations and MS analysis of the serum *N-*glycome

Samples were analyzed using a microfluidic chip-LC coupled with the Agilent 6550 iFunnel Accurate Mass Quadrupole Time-of-Flight Mass Spectrometer System (Agilent Technologies, Santa Clara, CA, USA) equipped with an auto-sampler (maintained at 5 °C), capillary pumps, nano pumps, and a chip/MS interface. The microfluidic chip consisted of a 40-nL enrichment column and a 43 × 0.075 mm i.d. analytical column, both packed with 5 μm graphitized carbon as the stationary phase, with integrated nano-ESI spray tips. For each sample, 1.0 μL of sample solution was loaded onto the enrichment column and washed with a solution of 0.1% formic acid (v/v) in water. A rapid *N-*glycan elution gradient was delivered at 0.5 μL/min using solutions of (A) 65 mM formic acid buffered to pH 3 in water and (B) 100% acetonitrile, at the following proportions and time points: 5–60%, 0–12 min; Remaining non-glycan compounds were flushed out with 80% B at 0.5 μL/min for 3 min, while the enrichment column was re-equilibrated with 0.1% formic acid at 3 μL/min for 10 min. The drying gas temperature was set at 225 °C with a flow rate of 11 L/min (filtered nitrogen gas). MS spectra were acquired over a mass range of *m*/*z* 500–3000 with an acquisition time of 1.0 s per spectrum in positive ionization mode. The instrument was operated using the target MS/MS mode, with the *m/z* range from *m*/*z* 50 to 3000 with an acquisition time of 1.5 s per spectrum.

Mass correction was enabled using a reference mass of *m*/*z* 922.0098 as the internal standard (G1969-85001; Agilent Technologies). The collision energy was set at 5–20 V. The full-width half maximum of the quadrupole mass bandpass used during MS/MS precursor isolation was set to medium (~4 *m*/*z*).

### LC/MS data processing and *N-*glycan identification using accurate mass data

LC/MS raw data were processed using the Molecular Feature Extractor (MFE) algorithm (Version B.06.00; Agilent Technologies). MFE is able to perform chemical relationship testing and chromatographic covariance testing, identify charge carriers (such as sodium) and multimers and group them, and reconstruct spectra by including isotope information. The critical parameters setting in the MFE includes the *N-*glycan model in the isotope model item, three as the maximum charge states, and 20 ppm as the accurate mass criteria. MS peaks were filtered with a signal-to-noise ratio of 5.0 and parsed into individual ion species. Using the expected isotopic distribution, charge state information and retention time, all ion species associated with a single compound (*e.g.*, the doubly protonated ion, the triply protonated ion, and all associated isotopologues) were totaled, and the neutral monoisotopic mass of the compound was calculated. Using this information, a list of all peaks in the sample was generated with abundances represented by chromatographic peak areas. Computerized algorithms were used to identify *N-*glycan compositions by accurate mass. By combining these empirical findings with previous research into the *N-*glycans of mammals, a virtual personal compound database and library (PCDL) was established resulting in over 4000 *N-*glycan compositions, which contained all biologically plausible rat serum *N-*glycan compositions with modifications such as *O-*acetylation, methylation, lactylation, sulfation, phosphorylation, and glycosylation. Deconvoluted experimental masses were compared against theoretical *N-*glycan masses using a mass error tolerance of 5 ppm. The scoring of the generated formulas was based on three factors: first, the measured mass (or *m/z*) was compared with the value predicted from the proposed formula; second, the abundance pattern of the measured isotope cluster was compared with values predicted from the proposed formula; third, the *m/z* spacing between the lowest *m/z* ion and the A + 1 and A + 2 ions were compared with the values predicted from the proposed formula. These individual factors were computed as match probabilities. Combining the individual match probabilities into an overall score was done as a weighted average rather than as a product. On the basis of known rat serum *N-*glycosylation patterns, *N-*glycan compositions containing hexose (Hex), *N-*acetylhexosamine (HexNAc), deoxyhexose (dHex), *N-*acetylneuraminic acid (NeuAc), *N-*glycolylneuraminic acid (NeuGc), and *O-*acetylation (OAc) were considered.

## Additional Information

**How to cite this article**: Gao, W.-N. *et al.* Microfluidic Chip-LC/MS-based Glycomic Analysis Revealed Distinct *N*-glycan Profile of Rat Serum. *Sci. Rep.* **5**, 12844; doi: 10.1038/srep12844 (2015).

## Supplementary Material

Supplementary Information

## Figures and Tables

**Table 1:**
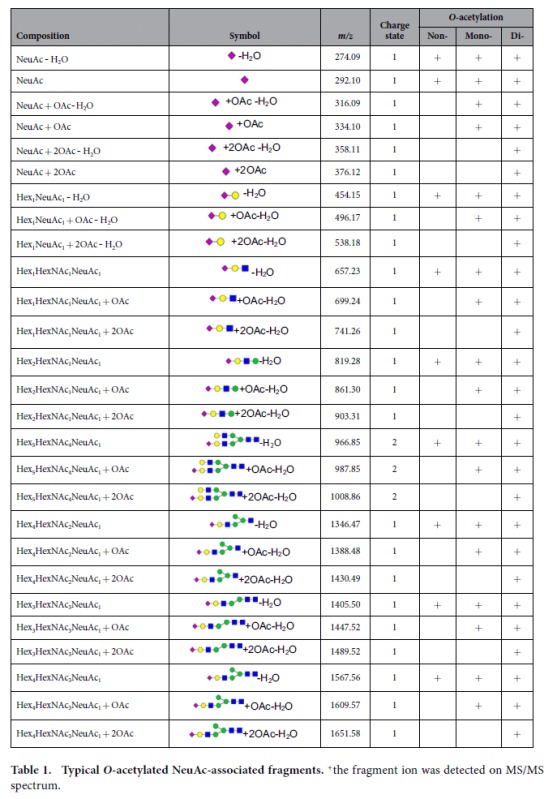
Typical *O-*acetylated NeuAc-associated fragments.

^+^the fragment ion was detected on MS/MS spectrum.

**Table 2:** Identified *N-*glycans containing *O-*acetylated sialic acid.

No.	Name	Abbr. *N-*glycan Composition	Molecular Formula	Calculated Mass	Observed Mass	Error (ppm)	*t*_R_ (min)
1^&^	Hex_4_HexNAc_3_NeuAc_1_ + OAc	4_3_0_1^*a^	C61 H100 N4O45	1608.5660	1608.5637	−1.40	8.40, 9.87
2^&^	Hex_4_HexNAc_3_NeuAc_1_ + 2OAc	4_3_0_1^*b^	C63 H102 N4 O46	1650.5765	1650.5662	−6.27	8.14
3^&^	Hex_4_HexNAc_4_NeuAc_1_ + OAc	4_4_0_1^*a^	C69 H113 N5 O50	1811.6453	1811.6445	−0.48	7.68, 8.42
4	Hex_5_HexNAc_4_NeuAc_1_ + OAc	5_4_0_1^*a^	C75 H123 N5 O55	1973.6982	1973.6993	0.60	7.68, 7.98, 8.72, 9.40
5^&^	Hex_4_HexNAc_5_NeuAc_1_ + OAc	4_5_0_1^*a^	C77 H126 N6 O55	2014.7247	2014.726	0.43	9.75
6	Hex_5_HexNAc_3_NeuAc_2_ + OAc	5_3_0_2^*a^	C78 H127 N5 O58	2061.7142	2061.7202	−2.53	6.65
7^&^	Hex_5_HexNAc_4_dHex_1_NeuAc_1_ + OAc	5_4_1_1^*a^	C81 H133 N5 O59	2119.7561	2119.7595	−0.01	8.75, 9.35, 9.87
8^&^	Hex_5_HexNAc_4_dHex_1_NeuAc_1_ + 2OAc	5_4_1_1^*b^	C83 H135 N5 O60	2161.7666	2161.7697	1.42	8.60, 9.20
9	Hex_5_HexNAc_4_NeuAc_2_ + OAc	5_4_0_2^*a^	C86 H140 N6 O63	2264.7936	2264.7934	−0.08	7.93, 8.47, 8.80, 9.12, 9.40
10^&^	Hex_5_HexNAc_4_NeuAc_1_NeuGc_1_ + OAc	5_4_0_2^*#a^	C86 H140 N6 O64	2280.7885	2280.7874	−0.50	9.02
11	Hex_5_HexNAc_4_NeuAc_2_ + 2OAc	5_4_0_2^*b^	C88 H142 N6 O64	2306.8042	2306.8022	−0.83	8.34, 8.94, 9.52, 10.12
12^&^	Hex_5_HexNAc_5_dHex_1_NeuAc_1_ + OAc	5_5_1_1^*a^	C89 H146 N6 O64	2322.8354	2322.8427	3.11	9.15
13	Hex_5_HexNAc_4_NeuAc_2_ + 3OAc	5_4_0_2^*c^	C90 H144 N6 O65	2348.8147	2348.8135	−0.50	9.19, 9.39, 9.64, 9.80, 10.04, 10.22, 10.41
14	Hex_5_HexNAc_5_dHex_1_NeuAc_1_ + 2OAc	5_5_1_1^*b^	C91 H148 N6 O65	2364.8460	2364.8431	−1.25	9.48, 10.10,
15	Hex_5_HexNAc_4_dHex_1_NeuAc_2_ + OAc	5_4_1_2^*a^	C92 H150 N6 O67	2410.8515	2410.8511	−0.14	8.39, 8.69, 8.97, 9.20, 9.70, 9.85, 10.37, 10.64
16^&^	Hex_5_HexNAc_4_dHex_1_NeuAc_1_NeuGc_1_ + OAc	5_4_1_2^*#a^	C92 H150 N6 O68	2426.8464	2426.8349	−4.75	8.67, 8.89
17^&^	Hex_5_HexNAc_4_dHex1NeuAc_2_ + 2OAc	5_4_1_2^*b^	C94 H152 N6 O68	2452.8620	2452.8623	0.12	9.35, 9.57, 9.94, 10.42, 10.84, 11.59
18^&^	Hex_5_HexNAc_5_NeuAc_2_ + OAc	5_5_0_2^*a^	C94 H153 N7 O68	2467.8729	2467.8555	−7.06	11.02
19^&^	Hex_5_HexNAc_4_dHex_1_NeuAc_2_ + 3OAc	5_4_1_2^*c^	C96 H154 N6 O69	2494.8926	2494.8699	−1.10	9.82, 10.00, 10.31, 10.49, 10.67
20	Hex_5_HexNAc_4_NeuAc_3_ + OAc	5_4_0_3^*a^	C97 H157 N7 O71	2555.8890	2555.8877	−0.51	8.85, 9.25, 9.75, 10.00, 10.46
21	Hex_5_HexNAc_4_NeuAc_3_ + 2OAc	5_4_0_3^*b^	C99 H159 N7 O72	2597.8996	2597.9010	0.57	9.05, 9.77, 10.19, 10.57, 11.19, 11.77
22	Hex_5_HexNAc_4_NeuAc_3_ + 3OAc	5_4_0_3^*c^	C101 H161 N7 O73	2639.9101	2639.9032	−2.61	9.94, 10.27, 10.79, 11.31, 12.01
23	Hex_5_HexNAc_4_dHex1NeuAc_3_ + OAc	5_4_1_3^*a^	C103 H167 N7 O75	2701.9469	2701.9497	1.02	9.85, 10.22, 10.84
24	Hex_5_HexNAc_4_dHex1NeuAc_3_ + 2OAc	5_4_1_3^*b^	C105 H169 N7 O76	2743.9575	2743.9537	−1.39	10.07, 10.32, 10.51, 10.96, 11.47
25	Hex_5_HexNAc_4_dHex1NeuAc_3_ + 3OAc	5_4_1_3^*c^	C107 H171 N7 O77	2785.9680	2785.9679	−0.04	10.32, 10.79, 11.11, 11.59, 12.46
26	Hex_6_HexNAc_5_NeuAc_3_ + OAc	6_5_0_3^*a^	C111 H180 N8 O81	2921.0212	2921.0160	−1.77	10.0, 10.37, 10.81, 11.27, 11.46
27	Hex_6_HexNAc_5_NeuAc_3_ + 2OAc	6_5_0_3^*b^	C113 H182 N8 O82	2963.0318	2963.0250	−2.28	10.32, 10.72, 11.02, 11.44, 12.07
28	Hex_6_HexNAc_5_NeuAc_3_ + 3OAc	6_5_0_3^*c^	C115 H184 N8 O83	3005.0423	3005.0390	−1.10	11.42
29	Hex_6_HexNAc_5_NeuAc_4_ + 2OAc	6_5_0_4^*b^	C124 H199 N9 O90	3254.1272	3254.1221	−1.57	11.29, 11.77
30	Hex_6_HexNAc_5_NeuAc_4_ + 3OAc	6_5_0_4^*c^	C126 H201 N9 O91	3296.1378	3296.1379	0.06	11.49, 11.96

^&^novel *N-*glycan; *NeuAc; ^#^NeuGc; ^a^mono-*O-*acetylation; ^b^bi-*O-*acetylation; ^c^tri-*O-*acetylation.
