# Supplementary material for: Microfluidic Chip-LC/MS-based Glycomic Analysis Revealed Distinct N-glycan Profile of Rat Serum
Source: Sci Rep. 2015 Aug 7;5:12844. doi: 10.1038/srep12844 (PMC4650694; doi:10.1038/srep12844)
Supplement: Supplementary Information [file srep12844-s1.doc]

**Supplementary information**

**Microfluidic Chip-LC/MS-based Glycomic Analysis Revealed Distinct N-glycan Profile of Rat Serum**

Wei-Na Gao^1^, Lee-Fong Yau^1^, Liang Liu^1^, Xing Zeng^2^, Da-Can Chen^2^, Min Jiang^3^, Ju Liu^3^, Jing-Rong Wang^1*^, Zhi-Hong Jiang^1*^

^1^ State Key Laboratory of Quality Research in Chinese Medicines, Macau Institute for Applied Research in Medicine and Health, Macau University of Science and Technology, Macau, China

^2^ Guangdong Provincial Hospital of Chinese Medicine, Second Clinical College of Guangzhou University of Chinese Medicine, Guangdong Provincial Academy of Chinese Medical Sciences, Guangzhou, China

^3^ Division of Rheumatology, Jiujiang First People's Hospital, Jiujiang, China;

*Correspondence and requests for materials should be addressed to J.-R. Wang (email: jrwang@must.edu.mo) and Z.-H. Jiang (email: zhjiang@must.edu.mo).

**FigureS1. Selection of digestion method for the release of *N*-glycans from serum glycoproteins.** Intensities of all acidic (containing sialic acid) and neutral (without sialic acid) *N*-glycans (a), and six randomly selected individual *N*-glycans (b). Each bar represents mean ± SD (*n* = 3).

**FigureS2. Selection of enrichment method for *N*-glycans.** Intensities of all acidic (containing sialic acid) and neutral (without sialic acid) *N*-glycans (a), and six randomly selected individual *N*-glycans (b). Each bar represents mean ± SD (*n* = 3).

**FigureS3. Optimization of mobile phase for highly-sialylated *N*-glycans.**

**FigureS4. The influence of the terminal sugar on the retention behavior of *N*-glycans on the PGC chip.** Elution of *N*-glycans with varying degrees of galactosylation (a), sialylation (b), and fucosylation(c).

**FigureS5. Separation of isomeric species of *N*-glycans on PGC chip.**

**FigureS6. Base peak chromatographs (BPC) of the *N*-glycans from repeated injections (*n* = 6) (a) and of the *N*-glycans from the sera of six rats (b).**

**TableS1. *N*-glycans identified in rat, mouse, and human sera.**

**
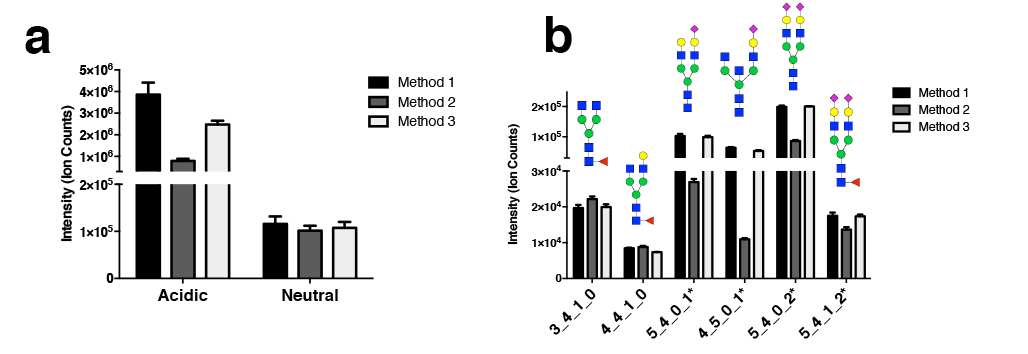
**

**Figure S1**

**
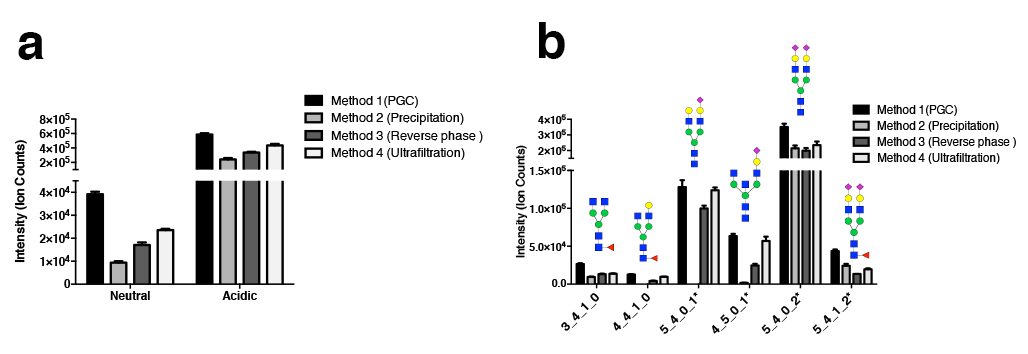
Figure S2**


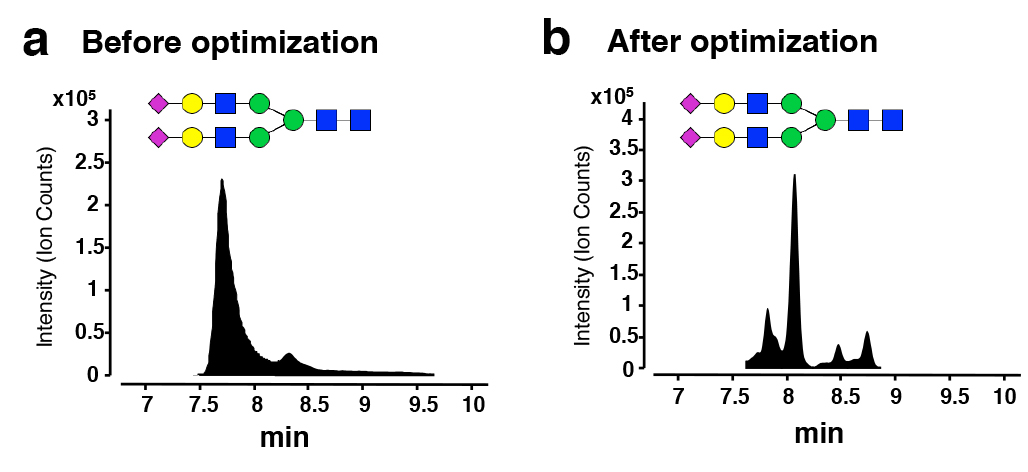


**Figure S3**


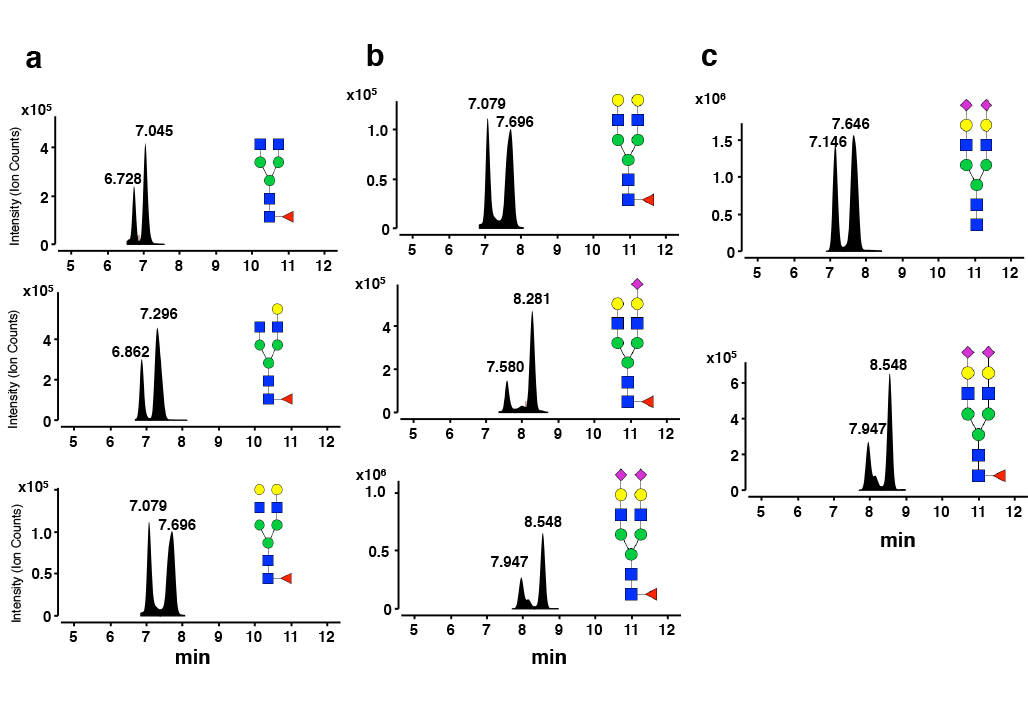


**Figure S4**

**Figure S5**

**
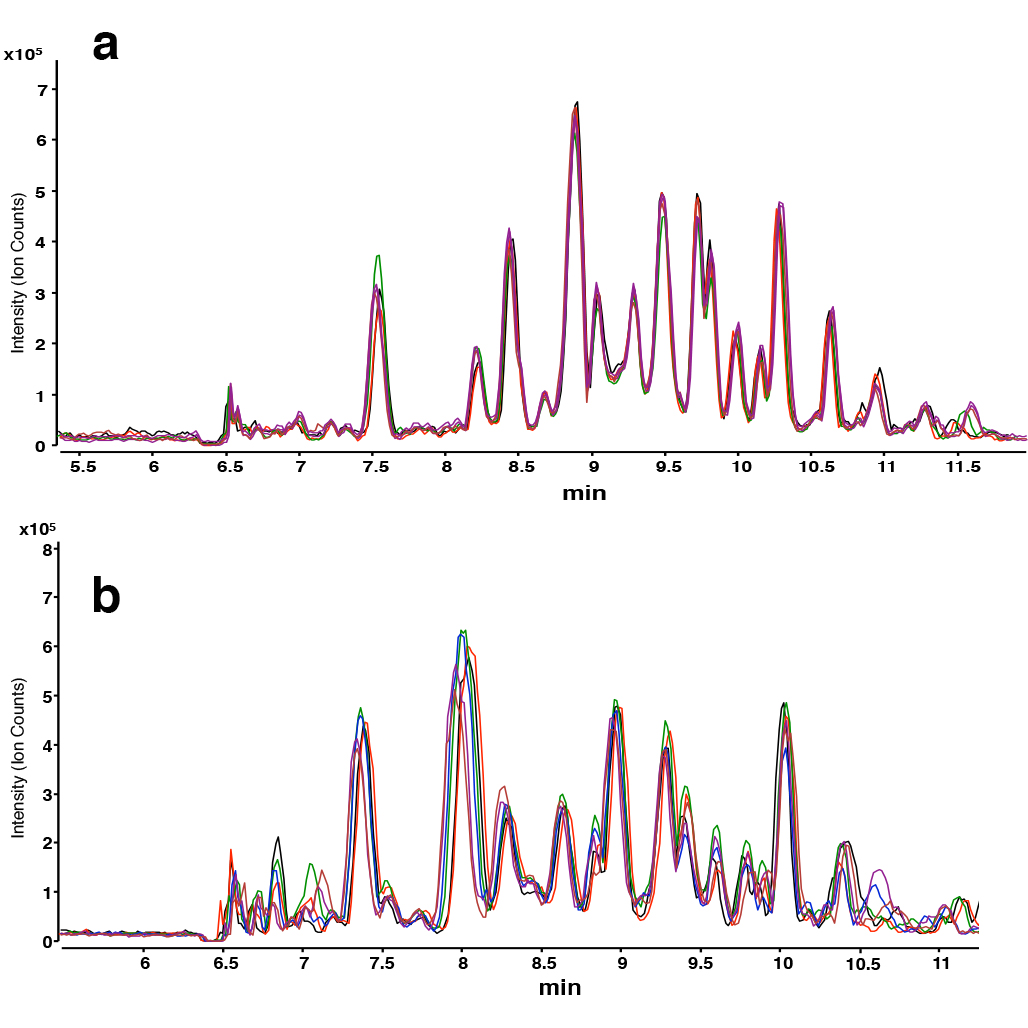
**

**FigureS6**

TableS1

| **No.** | **Full name** | **Abbreviation** | **Molecular**  **formula** | **Measuredmass** | **Calculatedmass** | **Error**  **(ppm)** | ***t*_R_**  **(min)** | **Fragment**  **(*m/z*)** | **Rat**  **serum** | **Human**  **serum** | **Mouse**  **serum** |
| --- | --- | --- | --- | --- | --- | --- | --- | --- | --- | --- | --- |
| 1 | Hex_1_HexNAc_2_ | 1_2_0_0 | C_22_H_38_N_2_O_16_ | 586.2215 | 586.2221 | -1.13 | 6.58,6.71,7.47,7.83 | 366.1308.204.0857 | + | + | + |
| 2 | Hex_2_HexNAc_2_ | 2_2_0_0 | C_28_H_48_N_2_O_21_ | 748.2684 | 748.2750 | -8.72 | 6.60,6.70,7.10,7.45,7.82,8.15 | 587.2293,528.1955,366.1401,204.0866 | + | + | + |
| 3 | Hex_3_HexNAc_2_ | 3_2_0_0 | C_34_H_58_N_2_O_26_ | 910.3277 | 910.3278 | -0.13 | 6.58,6.72,6.92,7.47,7.82 | 749.2805,528.1952,366.1393,204.0856 | + | + | + |
| 4 | Hex_3_HexNAc_2_dHex_1_ | 3_2_1_0 | C_40_H_68_N_2_O_30_ | 1056.3740 | 1056.3857 | -11.02 | 6.73 | 366.1379,204.0872 |  | + |  |
| 5 | Hex_4_HexNAc_2_ | 4_2_0_0 | C_40_H_68_N_2_O_31_ | 1072.3780 | 1072.3806 | -2.41 | 6.58,6.72,6.9,7.03,7.47,7.97 | 911.3276,749.2792,528.1914,425.1715,366.1319,204.0887 | + | + | + |
| 6 | Hex_3_HexNAc_3_ | 3_3_0_0 | C_42_H_71_N_3_O_31_ | 1113.4019 | 1113.4072 | -4.70 | 6.70,7.10,7.32,7.85 | 951.7778,911.3217,749.2918,690.2067,366.1416,204.0897 | + | + | + |
| 7 | Hex_5_HexNAc_2_ | 5_2_0_0 | C_46_H_78_N_2_O_36_ | 1234.4326 | 1234.4334 | -0.66 | 6.58,6.72,6.90,7.47,7.83 | 1217.4233,1073.3805,1014.3418,749.2893,690.2472,587.32375,528.1960,366.1363,204.0822 | + | + | + |
| 8 | Hex_3_HexNAc_3_dHex_1_ | 3_3_1_0 | C_48_H_81_N_3_O_35_ | 1259.4644 | 1259.4651 | -0.54 | 6.73,7.12 | 1098.4259,1057.3960,911.3148,749.3016,690.2545,528.1933,366.1423,204.0883 | + | + |  |
| 9 | Hex_4_HexNAc_3_ | 4_3_0_0 | C_48_H_81_N_3_O_36_ | 1275.4576 | 1275.4600 | -1.88 | 6.62,6.80,7.08 | 366.1396,204.0866 | + | + | + |
| 10 | Hex_3_HexNAc_4_ | 3_4_0_0 | C_50_H_84_N_4_O_36_ | 1316.4875 | 1316.4865 | 0.75 | 6.57,6.7,7.08 | 1114.4107,952.5247,893.3200,366.1349,204.0856 | + | + | + |
| 11 | Hex_6_HexNAc_2_ | 6_2_0_0 | C_52_H_88_N_2_O_41_ | 1396.4854 | 1396.4863 | -0.64 | 6.58,6.72,7.08 | 1235.4363,1176.4067,1073.4022,1014.3397,690.2495,366.1437,204.0830 | + | + | + |
| 12 | Hex_4_HexNAc_3_dHex_1_ | 4_3_1_0 | C_54_H_91_N_3_O_40_ | 1421.514 | 1421.5179 | -2.75 | 7.38 | 366.1398,204.0853 | + | + |  |
| 13 | Hex_5_HexNAc_3_ | 5_3_0_0 | C_54_H_91_N_3_O_41_ | 1437.508 | 1437.5128 | -3.36 | 6.68,7.03 | 366.1407,204.0858 | + | + | + |
| 14 | Hex_3_HexNAc_4_dHex_1_ | 3_4_1_0 | C_56_H_94_N_4_O_40_ | 1462.5454 | 1462.5444 | 0.06 | 6.75,7.10 | 1317.5589,1114.4065,1098.4211,893.3197,690.8829,366.1444,204.0873 | + | + | + |
| 15 | Hex_4_HexNAc_4_ | 4_4_0_0 | C_56_H_94_N_4_O_41_ | 1478.5355 | 1478.5393 | -2.61 | 6.62,6.80 | 1290.4968,528.1790,366.1434,204.0873 |  | + | + |
| 16 | Hex_3_HexNAc_5_ | 3_5_0_0 | C_58_H_97_N_5_O_41_ | 1519.5522 | 1519.5659 | -9.05 | 6.52 | 366.1411,204.0876 |  | + |  |
| 17 | Hex_7_HexNAc_2_ | 7_2_0_0 | C_58_H_98_N_2_O_46_ | 1558.5377 | 1558.5391 | -0.89 | 6.39,6.98 | 1397.4531,1235.4251,1073.2552,528.1836,505.1087,366.1333,204.0854 | + | + | + |
| 18 | Hex_4_HexNAc_3_NeuAc_1_ | 4_3_0_1^*^ | C_59_H_98_N_4_O_44_ | 1566.5549 | 1566.5554 | -0.30 | 6.42,6.82,7.23,8.04,8.62 | 1258.4949,1143.363,1114.4213,911.3316,893.3448,819.2906,749.2824,731.2721,657.2345,366.1456,292.0973,274.0964,204.0829 | + | + |  |
| 19 | Hex_4_HexNAc_3_NeuGc_1_ | 4_3_0_1^#^ | C_59_H_98_N_4_O_45_ | 1582.5453 | 1582.5503 | -3.17 | 7.43 | 1276.4676,911.3336,893.3367,749.2713,690.2457,366.1375,316.0751,294.0774,204.0872 | + |  | + |
| 20 | Hex_5_HexNAc_3_dHex_1_ | 5_3_1_0 | C_60_H_101_N_3_O_45_ | 1583.5668 | 1583.5707 | -2.45 | 6.95 | 366.1344,204.0878 |  | + |  |
| 21 | Hex_6_HexNAc_3_ | 6_3_0_0 | C_60_H_101_N_3_O_46_ | 1599.5621 | 1599.5656 | -2.21 | 6.81,7.37 | 1420.5942,1379.5891,1235.4337,1218.1225,1194.2884,1176.4508,1114.3538,1096.6411,1073.3714,1055.4539,528.1441,505.1096,487.1367,425.1673,366.1415,204.0887 | + | + | + |
| 22 | Hex_4_HexNAc_3_NeuAc_1_+OAc | 4_3_0_1^*a^ | C_61_H_100_N_4_O_45_ | 1608.5637 | 1608.566 | -1.40 | 8.40,9.87 | 1388.5094,1096.4115,911.3334,699.2423,366.1399,334.1129,316.1055,292.1018,274.0913,204.0084 | + |  |  |
| 23 | Hex_4_HexNAc_4_dHex_1_ | 4_4_1_0 | C_62_H_104_N_4_O_45_ | 1624.5969 | 1624.5973 | -0.20 | 6.58,6.88,7.37 | 1422.5221,1260.4611,1242.4481,1055.3821,571.2302,366.1389,204.0853 | + | + | + |
| 24 | Hex_5_HexNAc_4_ | 5_4_0_0 | C_62_H_104_N_4_O_46_ | 1640.5902 | 1640.5922 | -1.21 | 6.71,7.0 | 1461.7507,1438.5384,1420.4893,1276.4631,1258.456,1096.7011,366.1418,204.0082 |  | + | + |
| 25 | Hex_4_HexNAc_3_NeuAc_1_+2OAc | 4_3_0_1^*b^ | C_63_H_102_N_4_O_46_ | 1650.5662 | 1650.5765 | -6.27 | 8.14 | 699.2412,366.1373,204.0872 | + |  |  |
| 26 | Hex_3_HexNAc_5_dHex_1_ | 3_5_1_0 | C_64_H_107_N_5_O_45_ | 1665.6231 | 1665.6238 | -0.44 | 6.58 | 366.1439,204.0864 | + | + | + |
| 27 | Hex_4_HexNAc_5_ | 4_5_0_0 | C_64_H_107_N_5_O_46_ | 1681.6083 | 1681.6187 | -6.21 | 6.53 | 366.1477,204.0868 |  | + |  |
| 28 | Hex_4_HexNAc_3_dHex_1_NeuAc_1_ | 4_3_1_1^*^ | C_65_H_108_N_4_O_48_ | 1712.6007 | 1712.6133 | -7.34 | 7.65 | 274.0922,366.1392,204.0891 |  | + |  |
| 29 | Hex_8_HexNAc_2_ | 8_2_0_0 | C_64_H_108_N_2_O_51_ | 1720.592 | 1720.5919 | 0.05 | 6.58,6.80 | 1518.4566,1397.4765,1235.4385,1073.3853,649.2002,587.2465,526.1373,366.1445,325.1133,204.0833 | + | + | + |
| 30 | Hex_5_HexNAc_3_NeuAc_1_ | 4_3_1_1^#^ | C_65_H_108_N_4_O_49_ | 1728.609 | 1728.6082 | 0.43 | 6.73,7.02,7.33,7.73,7.97 | 1073.3831,1055.392,911.3362,819.286,784.2840(2+),755.2526(2+),719.7537(2+),710.2615(2+),703.2610(2+),657.2342,366.1406,292.1038,274.0927,204.0876 | + | + |  |
| 31 | Hex_6_HexNAc_3_dHex_1_ | 6_3_1_0 | C_66_H_111_N_3_O_50_ | 1745.6235 | 1745.6235 | -0.02 | 6.73,7.02,7.28,7.67 | 366.1428,204.0874 | + | + |  |
| 32 | Hex_7_HexNAc_3_ | 7_3_0_0 | C_66_H_111_N_3_O_51_ | 1761.6203 | 1761.6184 | 1.06 | 7 | 366.1389,204.0872 | + |  |  |
| 33 | Hex_4_HexNAc_4_NeuAc_1_ | 4_4_0_1^*^ | C_67_H_111_N_5_O_49_ | 1769.6312 | 1769.6348 | -2.03 | 6.93,7.38,8.39 | 1567.5625,1549.5585,1346.4712,1114.4243,1096.4123,952.361,934.3432,911.3344,819.296,740.2780(2+),731.2775(2+),657.2361,366.1404,292.1021,274.0897,204.0858 | + | + |  |
| 34 | Hex_4_HexNAc_4_dHex_2_ | 4_4_2_0 | C_68_H_114_N_4_O_49_ | 1770.6476 | 1770.6552 | -4.28 | 7.97 | 366.1357,204.0859 | + |  |  |
| 35 | Hex_4_HexNAc_4_NeuGc_1_ | 4_4_0_1^#^ | C_67_H_111_N_5_O_50_ | 1785.6372 | 1785.6297 | 4.19 | 6.53 | 690.237,673.2347,366.1372,308.0564,290.0343,204.0829 |  |  | + |
| 36 | Hex_5_HexNAc_4_dHex_1_ | 5_4_1_0 | C_68_H_114_N_4_O_50_ | 1786.6482 | 1786.6501 | -1.03 | 6.55,6.90,7.10,7.37,7.73 | 1420.5342,1404.5267,553.2225,528.1907,366.1373,204.0872 | + | + |  |
| 37 | Hex_6_HexNAc_4_ | 6_4_0_0 | C_68_H_114_N_4_O_51_ | 1802.658 | 1802.645 | 7.24 | 8.25 | 366.1396,204.0875 |  | + | + |
| 38 | Hex_4_HexNAc_4_NeuAc_1_+OAc | 4_4_0_1^*a^ | C_69_H_113_N_5_O_50_ | 1811.6445 | 1811.6393 | -0.48 | 7.68,8.42 | 1609.6062,1591.5538,1479.5204,699.2476,529.1902,496.1691,366.1381,334.1173,316.1015,204.0882 | + |  |  |
| 39 | Hex_4_HexNAc_5_dHex_1_ | 4_5_1_0 | C_70_H_117_N_5_O_50_ | 1827.6757 | 1827.6766 | -0.51 | 6.62,8.03 | 1625.602,1479.5308,1463.5252,841.8023(2+),731.2772(2+),366.1415,204.0886 | + | + | + |
| 40 | Hex_5_HexNAc_5_ | 5_5_0_0 | C_70_H_117_N_5_O_51_ | 1843.6915 | 1843.6715 | 10.8 | 7.18 | 1461.5388,832.7530(2+),740.2701(2+),719.7820(2+),546.2625,528.1096,366.1459,204.0906 |  | + |  |
| 41 | Hex_9_HexNAc_2_ | 9_2_0_0 | C_70_H_118_N_2_O_56_ | 1882.642 | 1882.6447 | -1.46 | 6.57 | 1459.4555,1397.5058,1037.4042,487.1772,366.1401,204.0887 | + |  | + |
| 42 | Hex_6_HexNAc_3_NeuAc_1_ | 6_3_0_1^*^ | C_71_H_118_N_4_O_54_ | 1890.661 | 1890.661 | -0.04 | 6.57,6.88,7.08,7.33,8.19 | 1582.5118,865.2773(2+),800.7943(2+),703.2499(2+),657.2293,407.1707,366.1359,292.1038,274.0872,204.0862 | + | + |  |
| 43 | Hex_4_HexNAc_4_dHex_1_NeuAc_1_ | 4_4_1_1^*^ | C_73_H_121_N_5_O_53_ | 1915.6847 | 1915.6927 | -4.14 | 7.93,8.30 | 657.2378,274.0931,366.1371,204.0896 | + | + |  |
| 44 | Hex_5_HexNAc_4_NeuAc_1_ | 5_4_0_1^*^ | C_73_H_121_N_5_O_54_ | 1931.6882 | 1931.6876 | 0.34 | 6.60,7.08,7.43,7.70,8.25,8.67,9.07,9.39 | 1729.5861,1567.5455,1405.4995,1276.4807,911.3284,819.3035,812.1607(2+),731.2648(2+),657.2358,528.1868,366.1396,292.1041,274.0882,204.0866 | + | + |  |
| 45 | Hex_5_HexNAc_4_NeuGc_1_ | 5_4_0_1^#^ | C_73_H_121_N_5_O_55_ | 1947.6828 | 1947.6825 | 0.17 | 7.08,7.33,7.68 | 821.2938(2+),1583.5718,740.2734(2+),1276.4711,1258.4634,1114.4067,673.2265,366.1396,308.0713,290.0846,204.0844 |  |  | + |
| 46 | Hex_6_HexNAc_4_dHex_1_ | 6_4_1_0 | C_74_H_124_N_4_O_55_ | 1948.6985 | 1948.7079 | -2.25 | 8.15,8.49 | 800.7791(2+),699.2416(2+),366.1398,204.0857 |  | + |  |
| 47 | Hex_7_HexNAc_4_ | 7_4_0_0 | C_74_H_124_N_4_O_56_ | 1964.699 | 1964.6978 | 0.59 | 7.1 | 366.1362,204.0873 | + |  | + |
| 48 | Hex_4_HexNAc_5_NeuAc_1_ | 4_5_0_1^*^ | C_75_H_124_N_6_O_54_ | 1972.6943 | 1972.7141 | -10.17 | 6.8 | 1770.6384,1299.4276,1155.4024,837.2913,657.2266,454.1471,366.1371,292.1024,274.0947,204.0947 |  | + |  |
| 49 | Hex_5_HexNAc_4_NeuAc_1_+OAc | 5_4_0_1^*a^ | C_75_H_123_N_5_O_55_ | 1973.6993 | 1973.6922 | 0.6 | 7.68,7.98,8.72,9.40 | 1609.5654,1591.2996,1276.4477,1096.4072,699.2477,496.1555,425.1714,366.1398,334.1134,316.1004,292.1019,274.0931,204.0857 | + |  |  |
| 50 | Hex_5_HexNAc_5_dHex_1_ | 5_5_1_0 | C_76_H_127_N_5_O_55_ | 1989.7202 | 1989.7295 | -4.67 | 7.08 | 366.1441,204.0894 |  | + | + |
| 51 | Hex_6_HexNAc_5_ | 6_5_0_0 | C_76_H_127_N_5_O_56_ | 2005.7199 | 2005.7244 | -2.23 | 7.56 | 366.1360,204.0871 |  | + |  |
| 52 | Hex_4_HexNAc_5_NeuAc_1_+OAc | 4_5_0_1^*a^ | C_77_H_126_N_6_O_55_ | 2014.726 | 2014.7247 | 0.43 | 9.75 | 1429.4614,1119.9154,952.5851,731.7774(2+),699.2416,657.2391,366.6041,316.0668,292.0963,274.0898,204.0865 | + |  |  |
| 53 | Hex_10_HexNAc_2_ | 10_2_0_0 | C_76_H_128_N_2_O_61_ | 2044.5997 | 2044.6975 | 0.65 | 8.79 | 366.1374,204.0844 | + |  |  |
| 54 | Hex_6_HexNAc_3_dHex_1_NeuGc_1_ | 6_3_1_1 | C_77_H_128_N_4_O_59_ | 2052.7151 | 2052.7139 | 0.61 | 7.47 | 996.2243,673.2309,366.1351,204.0874 | + |  |  |
| 55 | Hex_5_HexNAc_3_NeuAc_2_+OAc | 5_3_0_2^*a^ | C_78_H_127_N_5_O_58_ | 2061.7142 | 2061.7202 | -2.53 | 6.65 | 699.2436,366.5989,316.1327,204.0882 | + |  |  |
| 56 | Hex_5_HexNAc_4_dHex_1_NeuAc_1_ | 5_4_1_1^*^ | C_79_H_131_N_5_O_58_ | 2077.7438 | 2077.7455 | -0.82 | 6.58,7.63,7.87,8.32,8.57,9.00,9.29,9.69 | 1713.6007,1533.5402,1422.5454,966.8662(2+),958.8410(2+),894.3434(2+),819.2709,657.2345,454.1559,366.1409,292.1097,274.0956,204.0874 | + | + |  |
| 57 | Hex_5_HexNAc_4_dHex_1_NeuGc_1_ | 5_4_1_1^#^ | C_79_H_131_N_5_O_59_ | 2093.7399 | 2093.7381 | -0.23 | 7.37,7.85,8.04,8.54,9.12,9.52 | 1729.6099,974.8544(2+),966.3406(2+),957.8163(2+),894.3441(2+),856.3102(2+),1567.5571,1422.5411,1404.5461,835.2846,673.2277,528.1885,366.1375,308.1071,290.0833,204.0875 | + |  | + |
| 58 | Hex_7_HexNAc_4_dHex_1_ | 7_4_1_0 | C_80_H_134_N_4_O_60_ | 2110.7513 | 2110.7557 | -2.1 | 7.08,8.92 | 1931.6688,1762.5483,1744.6437,1584.4694,1404.5132,975.6678(2+),366.1339,204.0861 | + |  | + |
| 59 | Hex_4_HexNAc_5_dHex_1_NeuAc_1_ | 4_5_1_1^*^ | C_81_H_134_N_6_O_58_ | 2118.7685 | 2118.772 | -1.68 | 6.52 | 1916.7174,949.9560(2+),657.2273,366.1388,292.0978,274.0895,204.0827 |  | + |  |
| 60 | Hex_5_HexNAc_4_dHex_1_NeuAc_1_+OAc | 5_4_1_1^*a^ | C_81_H_133_N_5_O_59_ | 2119.7595 | 2119.7561 | -0.01 | 8.75,9.35,9.87 | 1755.6285,987.8728(2+),894.8213(2+),877.3236(2+),1593.5856,702.2556,699.2472,528.1956,514.1726,366.1372,334.1136,316.1034,204.0862 | + |  |  |
| 61 | Hex_5_HexNAc_5_NeuAc_1_ | 5_5_0_1^*^ | C_81_H_134_N_6_O_59_ | 2134.7601 | 2134.7675 | -3.23 | 7.97 | 657.2269,274.0902,366.1363,204.0852 | + | + |  |
| 62 | Hex_6_HexNAc_5_dHex_1_ | 6_5_1_0 | C_82_H_137_N_5_O_60_ | 2151.7772 | 2151.7731 | -2.34 | 7.38 | 366.1387,204.0854 |  | + |  |
| 63 | Hex_5_HexNAc_4_dHex_1_NeuAc_1_+2OAc | 5_4_1_1^*a^ | C_83_H_135_N_5_O_60_ | 2161.7697 | 2161.7651 | 1.42 | 8.60,9.20 | 699.2416,366.1406,204.0884 | + |  |  |
| 64 | Hex_5_HexNAc_4_NeuAc_2_ | 5_4_0_2^*^ | C_84_H_138_N_6_O_62_ | 2222.7834 | 2222.783 | 0.17 | 6.72,7.58,7.87,8.24,9.07,9.65 | 1932.6956,1770.6439,1567.5630,1405.5055,1346.4670,1184.4147,966.8507(2+),856.3118(2+),819.2857,657.2338,472.1659,454.1529,366.1388,292.1021,274.0916,204.0860 | + | + |  |
| 65 | Hex_5_HexNAc_4_NeuAc_1_NeuGc_1_ | 5_4_0_2^*#^ | C_84_H_138_N_6_O_63_ | 2238.7781 | 2238.7793 | 0.09 | 6.58,7.52,7.87,8.15,9.04,9.64 | 1583.5583,1567.5632,1405.5125,981.3411,974.8490(2+),966.8537(2+),893.8225(2+),885.8252(2+),835.2989,819.2883,673.2307,657.2356,472.1679,454.1558,366.1435,308.0979,292.1033,290.0898,274.0951,204.0874 | + |  |  |
| 66 | Hex_5_HexNAc_4_NeuGc_2_ | 5_4_0_2^#^ | C_84_H_138_N_6_O_6_4 | 2254.7688 | 2254.7728 | -1.79 | 7.47,8.12 | 673.2289,366.1404,204.0862 | + |  | + |
| 67 | Hex_6_HexNAc_4_dHex_1_NeuGc_1_ | 6_4_1_1^#^ | C_85_H_141_N_5_O_64_ | 2255.7814 | 2255.7932 | -5.25 | 9.02 | 673.2355,366.1391,204.0876 | + |  |  |
| 68 | Hex_4_HexNAc_5_NeuAc_2_ | 4_5_0_2^*^ | C_86_H_141_N_7_O_62_ | 2263.806 | 2263.8096 | -1.55 | 7.17 | 966.8565,657.2249,454.1505,366.141,292.1015,274.087,204.0858 | + |  |  |
| 69 | Hex_5_HexNAc_4_NeuAc_2_+OAc | 5_4_0_2^*a^ | C_86_H_140_N_6_O_63_ | 2264.7934 | 2264.7937 | -0.08 | 7.93,8.47,8.80,9.12,9.40,10.01 | 1932.6994,1770.6435,1609.5752,1567.5626,1447.5236,1405.5214,1388.4801,1346.4667,987.8560(2+),966.8555(2+),861.2960,819.2910,699.2469,657.2359,496.1676,472.1701,454.1531,366.1395,334.1139,316.1039,292.1041,274.0931,274.0931,204.0869 | + |  |  |
| 70 | Hex_5_HexNAc_4_NeuAc_1_NeuGc_1_+OAc | 5_4_0_2^*#a^ | C_86_H_140_N_6_O_64_ | 2280.7874 | 2280.7885 | -0.5 | 9.02 | 1591.5632,1583.5575,987.8533(2+),965.8425(2+),906.8302(2+),893.8323(2+),877.3197(2+),861.2988,835.2832,819.2858,699.2458,673.2305,657.2348,528.1930,496.1668,366.1398,334.1140,316.0938,308.1012,292.1035,290.0980,274.0930,204.0871 | + |  |  |
| 71 | Hex_5_HexNAc_5_dHex_1_NeuAc_1_ | 5_5_1_1^*^ | C_87_H_144_N_6_O_63_ | 2280.8306 | 2280.8247 | 2.52 | 9.37 | 657.2319,274.0880,366.1379,204.0879 |  | + |  |
| 72 | Hex_6_HexNAc_5_NeuAc_1_ | 6_5_0_1^*^ | C_87_H_144_N_6_O_64_ | 2296.8203 | 2296.8198 | 0.21 | 10.15 | 657.2353,274.0843,366.1399,204.0886 |  | + |  |
| 73 | Hex_6_HexNAc_5_dHex_2_ | 6_5_2_0 | C_88_H_147_N_5_O_64_ | 2297.829 | 2297.8402 | -4.89 | 7.8 | 366.1358,204.8075 | + |  |  |
| 74 | Hex_5_HexNAc_4_NeuAc_2_+2OAc | 5_4_0_2^*b^ | C_88_H_142_N_6_O_64_ | 2306.8022 | 2306.8048 | -0.83 | 7.92,8.34,8.94,9.52,9.65,10.12 | 1932.6995,1651.5882,1609.5682,1567.5625,1489.5210,1447.5195,1430.4949,1405.5104,1388.4801,1346.4733,1008.8536(2+),987.8524(2+),966.8555(2+),903.3107,861.2978,819.2860,699.2456,657.2366,496.1662,454.1531,376.1174,366.1380,358.1121,334.1118,316.1022,292.0982,274.0931,204.0859 | + |  |  |
| 75 | Hex_5_HexNAc_5_dHex_1_NeuAc_1_+OAc | 5_5_1_1^*a^ | C_89_H_146_N_6_O_64_ | 2322.8427 | 2322.8354 | 3.11 | 9.15 | 1609.5712,1591.5616,1447.5133,987.8494(2+), 861.3237, 741.2549, 699.2425, 657.2389, 528.1792, 366.1389, 334.1094, 316.1022, 204.0863 | + |  |  |
| 76 | Hex_5_HexNAc_4_NeuAc_2_+3OAc | 5_4_0_2^*c^ | C_90_H_144_N_6_O_65_ | 2348.8135 | 2348.8147 | -0.5 | 9.19,9.39,9.64,9.80,10.04,10.22,10.41 | 1651.5744,1609.5706,1567.5611,1489.5315,1477.5210,1430.4944,1405.5109,1388.4804,1346.4733,903.3008,861.2938,819.2833,741.2548,699.2480,538.1839,496.1706,454.1561,358.1133,334.1091,316.1004,292.1026,274.0928,204.0865 | + |  |  |
| 77 | Hex_5_HexNAc_5_dHex_1_NeuAc_1_+2OAc | 5_5_1_1^*b^ | C_91_H_148_N_6_O_65_ | 2364.8431 | 2364.846 | -1.25 | 9.48,10.10 | 1674.5422,1651.575,1096.8421,1078.0389,757.2461,741.2608,699.2464,657.237,366.1443,334.1088,316.1091,272.1093,204.0894 | + |  |  |
| 78 | Hex_5_HexNAc_4_dHex_1_NeuAc_2_ | 5_4_1_2^+^ | C_90_H_148_N_6_O_66_ | 2368.8269 | 2368.8409 | -5.9 | 8.40,8.99,9.64,10.79 | 1550.5567,1112.8816(2+),1039.8941(2+),819.2873(2+),657.2369,528.1796,366.1369,292.1003,274.0895,204.0875 | + | + |  |
| 79 | Hex_5_HexNAc_4_dHex1NeuAc_1_NeuGc_1_ | 5_4_1_2^*#^ | C_90_H_148_N_6_O_67_ | 2384.8322 | 2384.8358 | -1.53 | 7.95,8.37,8.62,8.97 | 1185.4269(2+),1112.3950(2+),1039.8837(2+),966.8407(2+),958.8711(2+),819.2926,673.2524,657.2321,454.1663,366.1374,292.0987,274.0911,204.0858 | + |  |  |
| 80 | Hex_5_HexNAc_4_dHex_1_NeuGc_2_ | 5_4_1_2^#^ | C_90_H_148_N_6_O_68_ | 2400.8279 | 2400.8307 | -1.17 | 8.34,8.90 | 1085.6794(2+),1047.8760(2+),1038.8631(2+),673.2403,366.1368,308.0903,290.0844,204.0863 | + |  | + |
| 81 | Hex_5_HexNAc_4_dHex_1_NeuAc_2_+OAc | 5_4_1_2^*a^ | C_92_H_150_N_6_O_67_ | 2410.8511 | 2410.8465 | -0.14 | 8.39,8.69,8.97,9.20,9.70,9.85,10.37,10.64 | 1755.6241,1737.6016,1713.6193,1694.6065,1593.5771,1551.5576,1533.5452,1133.4079(2+),1124.3901(2+),1060.8784(2+),1051.8600(2+),1039.8672(2+),1030.8559(2+),979.3436(2+),958.8393(2+),861.2913,819.2724,699.2451,657.2325,496.1649,366.1379,334.1151,316.1015,292.1021,274.0889,204.0858 | + |  |  |
| 82 | Hex_5_HexNAc_5_NeuAc_2_ | 5_5_0_2^*^ | C_92_H_151_N_7_O_67_ | 2425.8621 | 2425.8624 | -0.11 | 9.85 | 1103.2374(2+),1608.5871,657.2311,454.1543,292.1058,274.0946,204.0876 |  | + |  |
| 83 | Hex_5_HexNAc_4_dHex_1_NeuAc_1_NeuGc_1_+OAc | 5_4_1_2^*#a^ | C_92_H_150_N_6_O_68_ | 2426.8349 | 2426.8495 | -4.75 | 8.67,8.89 | 1755.6253,1737.5998,1549.5554,1141.8700(2+),1060.8919(2+),1051.8668(2+),1039.8697(2+),970.3457(2+),861.2582,699.2457,673.2307,657.2312,496.1636,366.1397,350.1515,334.1140,316.1044,308.0967,292.1037,274.0895,204.0852 | + |  |  |
| 84 | Hex_5_HexNAc_5_NeuAc_1_NeuGc_1_ | 5_5_0_2^*#^ | C_92_H_151_N_7_O_68_ | 2441.8706 | 2441.8539 | 5.46 | 9.82 | 673.2195,657.2281,366.1362,204.0862 | + |  |  |
| 85 | Hex_6_HexNAc_5_dHex_1_NeuAc_1_ | 6_5_1_1^*^ | C_93_H_154_N_6_O_68_ | 2442.8763 | 2442.8777 | -0.57 | 7.35,7.9 | 657.2363,274.0881,366.1339,204.0813 |  | + |  |
| 86 | Hex_5_HexNAc_4_dHex_1_NeuAc_2_+2OAc | 5_4_1_2^*b^ | C_94_H_152_N_6_O_68_ | 2452.8623 | 2452.862 | 0.12 | 9.35,9.57,9.94,10.42,10.84,11.59 | 1593.5423,1575.5485,1154.3916(2+),1145.6619(2+),1060.8869(2+),979.8758(2+),878.8087(2+),699.2431,657.2387,496.1589,366.1363,334.1114,316.1050,292.0980,274.0903,204.0837 | + |  |  |
| 87 | Hex_5_HexNAc_5_NeuAc_2_+OAc | 5_5_0_2^*a^ | C_94_H_153_N_7_O_68_ | 2467.8555 | 2467.8729 | -7.06 | 11.02 | 1770.5633,1573.5076,1096.0526,879.3561,861.2992,819.2732,741.2534,699.2474,657.2366,366.1408,334.1121,316.1049,204.0844 | + |  |  |
| 88 | Hex_5_HexNAc_4_dHex_1_NeuAc_2_+3OAc | 5_4_1_2^*c^ | C_96_H_154_N_6_O_69_ | 2494.8699 | 2494.8623 | -1.1 | 9.82,10.00,10.31,10.49,10.67 | 1797.6300,1755.6417,1593.5623,1175.3980(2+),1166.4099(2+),1000.8507(2+),741.2526,699.2453,657.234,538.1776,366.1384,358.1108,334.1082,204.0864 | + |  |  |
| 89 | Hex_7_HexNAc_6_dHex_1_ | 7_6_1_0 | C_96_H_160_N_6_O_70_ | 2516.9159 | 2516.9145 | 0.057 | 8.52 | 1242.4737,893.3223,366.1372,204.0877 | + |  |  |
| 90 | Hex_5_HexNAc_4_NeuAc_3_+OAc | 5_4_0_3^*a^ | C_97_H_157_N_7_O_71_ | 2555.8877 | 2555.889 | -0.51 | 8.85,9.25,9.75,10.00,10.46 | 1840.3370,1609.5765,1591.5432,1168.3841(2+),1133.4097(2+),1124.8857(2+),1112.3942(2+),1075.9683(2+),1052.810(2+),1022.8786(2+),966.3450,948.3243,699.2458,657.2329,454.1508,407.8486,366.1388,334.1131,292.1019,274.0902,204.0850 | + |  |  |
| 91 | Hex_6_HexNAc_4_dHex_1_NeuGc_2_ | 6_4_1_2^#^ | C_96_H_158_N_6_O_73_ | 2562.8839 | 2562.8836 | 0.15 | 7.08,9.87 | 673.2213,366.1396,204.0859 | + |  |  |
| 92 | Hex_5_HexNAc_5_dHex_1_NeuAc_2_ | 5_5_1_2^*^ | C_98_H_161_N_7_O_71_ | 2571.8835 | 2571.9203 | -14.31 | 9.75 | 1204.3971(2+),1185.4419(2+),1133.4001(2+),1112.3922(2+),657.2321,366.1409,292.0986,274.0946,204.0856 |  | + |  |
| 93 | Hex_7_HexNAc_4_NeuGc_2_ | 7_4_0_2^#^ | C_96_H_158_N_6_O_74_ | 2578.8754 | 2578.8785 | -1.2 | 11.22 | 673.2188,366.1362,204.0875 | + |  |  |
| 94 | Hex_6_HexNAc_5_NeuAc_2_ | 6_5_0_2^*^ | C_98_H_161_N_7_O_72_ | 2587.9111 | 2587.9117 | -1.6 | 7.70,8.42,9.02,9.24,10.00 | 657.2361,274.0996,366.1350,204.0813 | + | + |  |
| 95 | Hex_5_HexNAc_5_dHex_1_NeuGc_2_ | 5_5_1_2^#^ | C_98_H_161_N_7_O_72_ | 2587.8955 | 2587.9152 | -7.61 | 10.34 | 657.2408,274.0897,366.1401,204.0888 | + |  |  |
| 96 | Hex_5_HexNAc_4_NeuAc_3_+2OAc | 5_4_0_3^*b^ | C_99_H_159_N_7_O_72_ | 2597.901 | 2597.8996 | 0.57 | 9.05,9.77,10.19,10.57,11.19,11.77 | 1152.3900,1290.9453(2+),1189.9072(2+),1032.3514,990.3415,861.2994,699.2414,657.2320,496.1646,366.143,351.0372,334.1115,316.0975,292.1037,274.0905,204.0858 | + |  |  |
| 97 | Hex_6_HexNAc_5_NeuAc_1_NeuGc_1_ | 6_5_0_2^*#^ | C_98_H_161_N_7_O_73_ | 2603.9181 | 2603.9101 | 3.07 | 6.58,7.23 | 673.2287,657.2377,366.1316,204.0875 | + |  |  |
| 98 | Hex_6_HexNAc_5_NeuGc_2_ | 6_5_0_2^#^ | C_98_H_161_N_7_O_74_ | 2619.8835 | 2619.905 | -8.21 | 9.79,10.20,10.57 | 673.2429,366.1424.204.0871 |  |  | + |
| 99 | Hex_4_HexNAc_9_dHex_1_ | 4_9_0_1 | C_102_H_169_N_9_O_70_ | 2639.9098 | 2639.91 | -11.96 | 10.70,11.21 | 366.1354,204.0876 | + |  |  |
| 100 | Hex_5_HexNAc_4_NeuAc_3_+3OAc | 5_4_0_3^*c^ | C_101_H_161_N_7_O_73_ | 2639.9032 | 2639.9101 | -2.61 | 9.94,10.27,10.79,11.31,12.01 | 1943.6614,1812.6227,1609.5681,1447.5094,1276.4622,1227.4585,1225.4144,1207.4295,1175.3943(2+),1154.4051(2+),1073.3926(2+),861.2977,819.2811,741.2442,699.2434,657.2339,528.1962,496.1644,425.1748,366.1383,348.0640,334.1119,316.1164,292.1009,204.0876 | + |  |  |
| 101 | Hex_5_HexNAc_4_dHex_1_NeuAc_3_+OAc | 5_4_1_3^*a^ | C_103_H_167_N_7_O_75_ | 2701.9497 | 2701.9469 | 1.02 | 9.85,10.22,10.84 | 1755.6421,1551.5666,1447.5123,1278.9555(2+),1206.4268(2+),1185.9294(2+),1125.3935(2+),1112.3700(2+),1057.3929,921.3125(2+),699.2462,657.2343,366.1397,334.1141,292.1015,274.0935,204.0836 | + |  |  |
| 102 | Hex_6_HexNAc_5_dHex_1_NeuAc2 | 6_5_1_2^*^ | C_104_H_171_N_7_O_76_ | 2733.964 | 2733.9731 | -3.35 | 9.95 | 657.2353,274.0893,366.1410,204.0877 |  | + |  |
| 103 | Hex_5_HexNAc_4_dHex_1_NeuAc_3_+2OAc | 5_4_1_3^*b^ | C_105_H_169_N_7_O_76_ | 2743.9537 | 2743.9575 | -1.39 | 10.07,10.32,10.51,10.96,11.47 | 1781.6036,1713.6133,1412.6809,1386.6627,1272.4127,1227.4274,1227.4585(2+),1154.4059(2+),1073.8903(2+),699.2410,657.2343,538.1945,496.1518,366.1389,334.1132,316.1048,292.1045,274.0902,204.0847 | + |  |  |
| 104 | Hex_5_HexNAc_4_dHex_1_NeuAc_3_+3OAc | 5_4_1_3^*c^ | C_107_H_171_N_7_O_77_ | 2785.9679 | 2785.968 | -0.04 | 10.32,10.79,11.11,11.59,12.46 | 1755.6238,1738.6287,1593.6158,1260.7416,1227.4089(2+), 1219.3267(2+), 1175.4249(2+), 699.2512, 657.2278, 538.2008, 366.1408, 334.1128, 316.1032, 292.1013, 204.0888 | + |  |  |
| 105 | Hex_6_HexNAc_5_NeuAc_3_ | 6_5_0_3^*^ | C_109_H_178_N_8_O_80_ | 2879.0219 | 2879.0106 | 3.94 | 8.55,8.95,9.29,9.79,10.22,10.62 | 1973.0383,1549.5487,657.2322,204.0814 | + |  |  |
| 106 | Hex_6_HexNAc_5_NeuAc_3_+OAc | 6_5_0_3^*a^ | C_111_H_180_N_8_O_81_ | 2921.016 | 2921.0212 | -1.77 | 10.00,10.37,10.81,11.27,11.46 | 2265.7921,1799.6366,1609.3223,948.3146(2+), 699.2498, 657.2373, 366.1391, 292.1087, 204.0856 |  | + |  |
| 107 | Hex_7_HexNAc_6_NeuAc_2_ | 7_6_0_2^*^ | C_112_H_184_N_8_O_82_ | 2953.0474 | 2953.0474 | -13.58 | 10.61 | 657.2354,274.0921,366.1357,204.0858 |  | + |  |
| 108 | Hex_6_HexNAc_5_NeuAc_3_+2OAc | 6_5_0_3^*b^ | C_113_H_182_N_8_O_82_ | 2963.025 | 2963.0318 | -2.28 | 10.32,10.72,11.02,11.44,12.07 | 2223.7264,1891.6633,1609.5657,1230.4294(2+),1184.9113, 1128.8815(2+), 699.2409, 657.2468, 587.2253, 528.1889, 366.1350,334.1140,316.0997,292.1011,274.0933,204.0900 | + |  |  |
| 109 | Hex_6_HexNAc_5_NeuAc_3_+3OAc | 6_5_0_3^*c^ | C_115_H_184_N_8_O_83_ | 3005.039 | 3005.0423 | -1.1 | 11.42 | 1812.6398,741.2608,699.2366,366.1397,358.1123,334.1157,316.1028,292.1027,274.0907,204.0884 | + |  |  |
| 110 | Hex_7_HexNAc_6_dHex_1_NeuAc_2_ | 7_6_1_2^*^ | C_118_H_194_N_8_O_86_ | 3099.0964 | 3099.1053 | -2.87 | 9.5 | 657.2284,274.0902,366.1347,204.0868 |  | + |  |
| 111 | Hex_7_HexNAc_6_NeuAc_3_ | 7_6_0_3^*^ | C_123_H_201_N_9_O_90_ | 3244.1449 | 3244.1428 | 0.63 | 8.54 | 657.2345,274.0943,366.1415,204.0875 |  | + |  |
| 112 | Hex_6_HexNAc_5_NeuAc_4_+2OAc | 6_5_0_4^*b^ | C_124_H_199_N_9_O_90_ | 3254.1221 | 3254.1272 | -1.57 | 11.29,11.77 | 1899.3332,990.3381,699.2372,657.2363,496.1606,454.1595,366.1421,334.1153,316.1066,292.1001,274.0941,204.0841 | + |  |  |
| 113 | Hex_6_HexNAc_5_NeuAc_4_+3OAc | 6_5_0_4^*c^ | C_126_H_201_N_9_O_91_ | 3296.1379 | 3296.1378 | 0.06 | 11.49,11.96 | 1380.9847(2+),1154.9293(2+),1124.3716(2+),699.2318,657.2324,366.1361,334.1162,316.1005,292.1136,204.1001 | + |  |  |
| 114 | Hex_7_HexNAc_6_dHex_1_NeuAc_3_ | 7_6_1_3^*^ | C_129_H_211_N_9_O_94_ | 3390.1884 | 3390.2007 | -3.63 | 13.78 | 657.2439,274.0895,366.1363,204.0866 |  | + |  |
| 115 | Hex7HexNAc6NeuAc4 | 7_6_0_4^*^ | C_134_H_218_N_10_O_98_ | 3535.2366 | 3535.2353 | -0.45 | 15.58 | 657.2392,274.0975,366.1431,204.0854 |  | + |  |
| 116 | Hex_7_HexNAc_6_dHex_1_NeuAc_4_ | 7_6_1_4^*^ | C_140_H_228_N_10_O_102_ | 3681.3019 | 3681.2961 | 1.56 | 10.42 | 657.2398,274.0904,366.1397,204.0813 |  | + |  |

^*^ NeuAc; **^#^** NeuGc; **^a^** mono-O-acetylation; **^b^** bi-O-acetylation; **^c^** bi-O-acetylation.
